# Assessing agreement between preclinical magnetic resonance imaging and histology: An evaluation of their image qualities and quantitative results

**DOI:** 10.1371/journal.pone.0179249

**Published:** 2017-06-30

**Authors:** Cindy Elschner, Paula Korn, Maria Hauptstock, Matthias C. Schulz, Ursula Range, Diana Jünger, Ulrich Scheler

**Affiliations:** 1 Leibniz Institute of Polymer Research Dresden, Dresden, Germany; 2 Department of Oral and Maxillofacial Surgery, Faculty of Medicine »Carl Gustav Carus«, Technische Universität Dresden, Dresden, Germany; 3 Institute for Medical Informatics and Biometry, Faculty of Medicine »Carl Gustav Carus«, Technische Universität Dresden, Dresden, Germany; Kyoto Daigaku, JAPAN

## Abstract

One consequence of demographic change is the increasing demand for biocompatible materials for use in implants and prostheses. This is accompanied by a growing number of experimental animals because the interactions between new biomaterials and its host tissue have to be investigated. To evaluate novel materials and engineered tissues the use of non-destructive imaging modalities have been identified as a strategic priority. This provides the opportunity for studying interactions repeatedly with individual animals, along with the advantages of reduced biological variability and decreased number of laboratory animals. However, histological techniques are still the golden standard in preclinical biomaterial research. The present article demonstrates a detailed method comparison between histology and magnetic resonance imaging. This includes the presentation of their image qualities as well as the detailed statistical analysis for assessing agreement between quantitative measures. Exemplarily, the bony ingrowth of tissue engineered bone substitutes for treatment of a cleft-like maxillary bone defect has been evaluated. By using a graphical concordance analysis the mean difference between MRI results and histomorphometrical measures has been examined. The analysis revealed a slightly but significant bias in the case of the bone volume $\left(bias_{Histo-MRI:Bone\;volume}=2.40\;\%,\;p<0.005\right)$ and a clearly significant deviation for the remaining defect width $(bias_{Histo-MRI:Defect\;width}=-6.73\;\%,\;p\ll 0.005).$ But the study although showed a considerable effect of the analyzed section position to the quantitative result. It could be proven, that the bias of the data sets was less originated due to the imaging modalities, but mainly on the evaluation of different slice positions. The article demonstrated that method comparisons not always need the use of an independent animal study, additionally.

## Introduction

The development of an optimal interface between biomaterial and tissue needs the confirmation of biocompatibility, safety and mechanical stability *in vitro* and *in vivo*. The extrapolation of *in vitro* results to the situation *in vivo* can be difficult because important factors like the complex tissue healing, status of bone, surgical technique and loading conditions cannot be simulated in the standardized environment. Therefore, the use of small-animal models in basic and preclinical science is indispensable for research and development strategies [1]. Analyzing the tissue response to biomaterials is mainly accomplished with histological methods [2,3]. However, these are destructive, time-consuming and lead to an information loss due to the unavoidable distances between sequenced slices which are the consequence of the cutting and grinding technique [4]. Moreover, histology consequently requires the animals to be sacrificed at each time point, making it impossible to study a single animal serially over time. According to *Appel et al*. and *Wehrli et al*. it is questionable in general whether few two-dimensional slices represent the whole three-dimensional and anisotropic bone architecture really exactly [2,5].

Hence, there is a great need to develop and evaluate high-resolution *in vivo* imaging technologies which allows repeated measurements with individual animals. Moreover, it has the advantage of a reduced biological variability and parallelly decreases the number of animals required for a study [6,7]. In principle, the same imaging modalities are available for preclinical research as those used in the clinical setting. Detailed, excellent reviews have been published which specified the different nondestructive imaging modalities for use in preclinical research [2,6–8].

Because the interface between implanted material and host system is the region of interest, and alternative imaging techniques have to compete with conventional histology which uses light microscopy, high-resolution imaging is of great importance. Ideally, histology and the preclinical imaging modalities obtain the same or comparable analytical parameters to describe the osseous situation. This is essential to find a broad acceptance within the biomaterial and medical community (*Parfitt et al*. give a detailed overview to referents and measurements used in bone histomorphometry [9]).

Assessing agreement between the image quality of histology and the alternative nondestructive imaging technique including also quantitative parameters is particularly important, but literature in biomaterial research is rare. For acceptance of non-destructive imaging in preclinical research the direct comparison of both the image qualities and quantitative results, especially in respect to histology, is indispensable because this is still the established golden standard.

The statistical approach to evaluate the degree of agreement of a measured quantity gained by two different techniques at the same individual or sample is called concordance analysis [10]. When comparing two methods of measurement, the difference between the measurement results (the »bias«) is the point of interest. A suitable and clear procedure is to use X-Y-scatter plots (X and Y refer to the methods of measurement) together with Bland-Altman diagrams which display the bias [11,12]. The decision to accept or reject the null hypothesis (»The measurement values do not distinguish themselves.«) can be taken by including the estimation of 95% confidence intervals (95% CI) for the bias like it was shown in the detailed review of *Giavarina* [13].

Within the present research paper a detailed concordance analysis is presented that assesses the degree of agreement between two imaging modalities. As an example, the possibility to use magnetic resonance imaging (MRI) to investigate the osseous integration of tissue engineered bone substitutes for the treatment of a cleft-like maxillary bone defect has been evaluated.

The incorporation of different tissue engineered grafts into the surrounding bone was analyzed by quantitative MRI and compared with conventional histology. Additionally, a contrasting juxtaposition of the image qualities of both MRI and histology was given.

Because the specimens analyzed here were obtained from an independent research project, no separate animal study was necessary [14].

### Clinical background—Clefts of the lip, alveolus and palate

Clefts of the lip, alveolus and palate (referred to as orofacial clefts) are birth defects that result from failure of fusion of the maxillary processes or palatal shelves during the early pregnancy [15]. Orofacial clefts occur in a wide geographic distribution and emerge at an average birth prevalence of 7.94 cleft lips per 10,000 live births [16]. The causes are complex, involving both genetic and environmental factors. Affected children need multidisciplinary care from birth until adulthood. Albeit the clinical treatment protocols for management of children with orofacial clefts are not consistent, several surgical procedures are always necessary [17,18]. Due to the fact that the alveolar osseous cleft is a critical size defect, a life-long nonunion would remain if untreated. An established procedure is the so-called secondary alveolar cleft osteoplasty in the mixed dentition phase with autologous bone grafting [18]. Augmenting the alveolar cleft with a bone substitute serves for multiple purposes: (1) it closes the oronasal fistulae and so gives bony support to the teeth adjacent to the cleft area, (2) furthermore, it allows the eruption of the permanent canine tooth, (3) it enables functional speech and swallowing, (4) and it is indispensable to restore the normal aesthetics and facial symmetry [14,19]. But the transplantation of autologous bone leads to another surgically created wound that might be accompanied with donor site morbidity [20]. Furthermore, the loss of the grafted bone due to infection or dehiscence of the wound is a frequently reported complication [18]. Additionally, the amount of transplantable bone is limited [21].

Therefore, artificial tissue constructs created by tissue engineering might be an interesting alternative to autogenous or allogenic grafts [22].

## Materials and methods

The present study was performed using tissue samples of an independent preclinical research project of the Faculty of Medicine »Carl Gustav Carus« (Department of Oral and Maxillofacial Surgery, Technische Universität Dresden, Dresden, Germany). The tissue engineering approach and details of the animal experiment have been also published by *Korn et al*. [14].

### Tissue engineering

#### Scaffold material

The commercially available and well established bone substitute of bovine origin (hydroxyapatite which includes purified porcine collagen; trade name: Bio-Oss^®^ collagen, Geistlich Pharma AG, Wollhusen, Switzerland) served as scaffold material [23]. The scaffolds are available as a small pad (size: 5.0 x 5.0 x 10 mm, á 100 mg) and were cropped into circular slices (diameter: 3.3 mm, thickness: 0.5 mm) according to the estimated defect size.

#### Tissue engineering

According to the experimental design two of the four groups were treated with tissue engineered bone graft substitutes (cf. Table 1). Mesenchymal stroma cells (MSCs) were isolated from the bone marrow of donor rats. Therefore, the femurs and tibia were extracted, the bone marrow punctured and the MSCs isolated. The cells were cultured in basic medium (MEM α, Gibco^®^, Life Technologies, Thermo Fisher Scientific Inc., Waltham, US) with 10% heat-inactivated fetal calf-serum (Gibco^®^, Life Technologies, Thermo Fisher Scientific Inc., Waltham, US) and 1% penicillin/streptomycin. One MSC fraction was cultivated in osteogenic differentiation medium (OPTIM-MEM, Gibco^®^, Life Technologies, Thermo Fisher Scientific Inc., Waltham, US) which included ascorbic acid, dexamethasone and β-glycerophosphate to differentiate osteoblasts from MSCs *in vitro*.

**Table 1:** Experimental design, all of the rats were assigned randomly to the experimental groups.

Group	Bone graft	*Healing time*					
		6 weeks		9 weeks		12 weeks	
1	Empty defect (control)	7^#^	2*	7^#^	2*	7^#^	3*
2	Bio-Oss^®^ collagen	7^#^	2*	7^#^	2*	7^#^	3*
3	Bio-Oss^®^ collagen, with MSCs	7^#^	2*	7^#^	2*	7^#^	3*
4	Bio-Oss^®^ collagen, with osteogenically differentiated MSCs	7^#^	2*	7^#^	2*	7^#^	3*

^#^ Overall number of laboratory animals

* Number of animals used for the concordance analysis

Loaded scaffolds were prepared using a cell suspension containing 200,000 MSCs or osteogenic differentiated MSCs, respectively.

### Animal experiment

#### Animal model and experimental design

All interventions were approved by the commission for animal studies at the district government Dresden, Germany (ethic vote No. 24(D)-9168.11-1/2013-7).

The study was performed using 84 male, adult Lewis rats (Janvier Labs, Le-Genest-Saint-Isle, France) with an average body weight of 460 g and an age of six to eight month at the beginning of the study. The animals have been housed in a light- and temperature-controlled environment with species-appropriate access to food and water *ad libitum*.

The animals were randomly assigned to the four experimental groups as listed in Table 1, whereof 28 animals were chosen arbitrarily for the concordance analysis (imaging with MRI and histology).

#### Animal surgery

**Fig 1:**
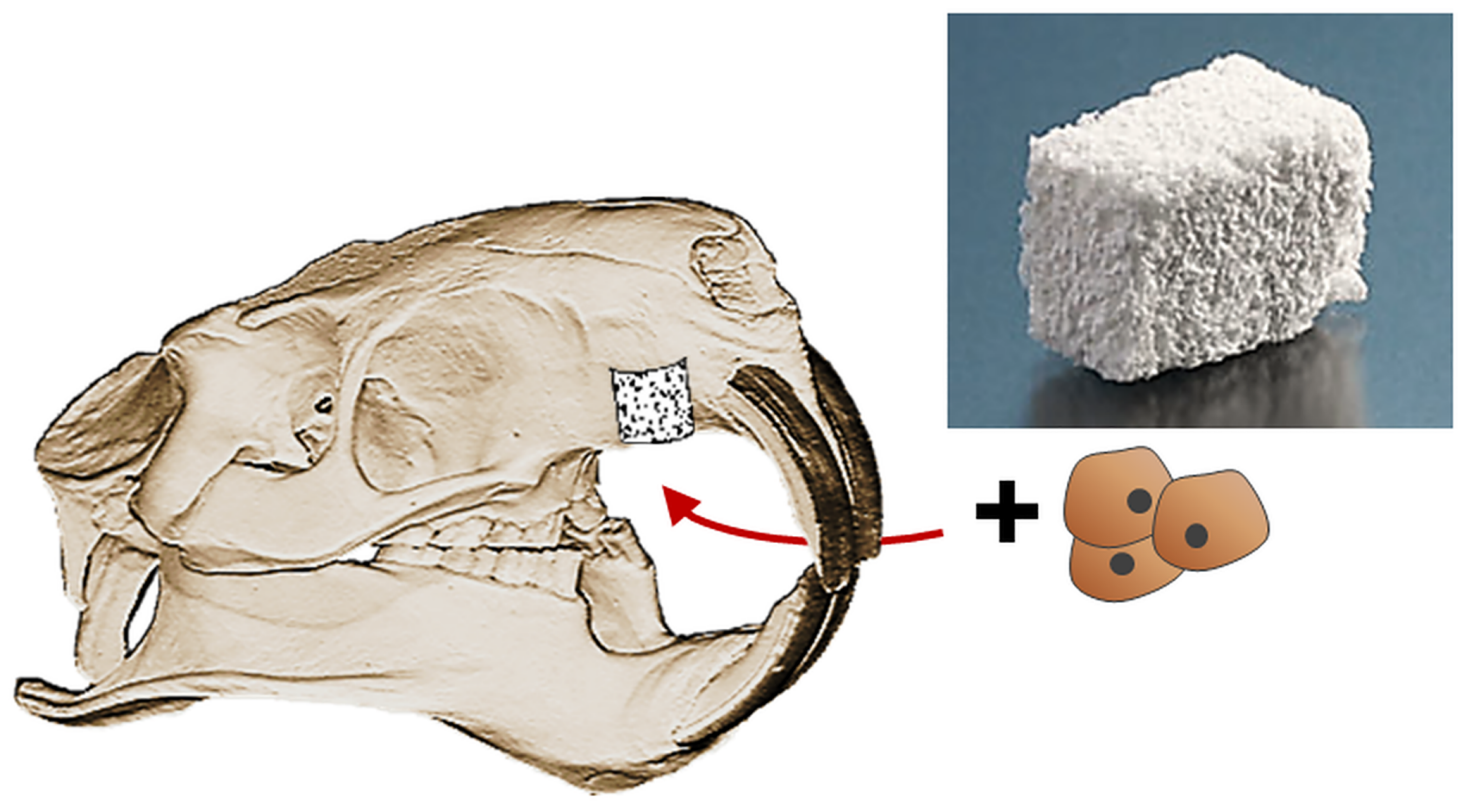
Augmenting the artificial, cylindrical, cleft-like defect with tissue engineered bone grafts.

The rats were anaesthetized with ketamine (ip injection, 100 mg/kg bw) and xylazine (ip injection, 10 mg/kg bw) and fixed in a dorsal position before creation of a simulated cleft-like defect in the anterior maxilla. For it, a sagittal incision following the mid-palatal suture was prepared. Then, a mucosal flap was elevated, the periosteum removed and a circular bone defect with a diameter of 3.3 mm created subsequently in the anterior maxilla (Fig 1).

According to the experimental design, each rat was randomly assigned to one experimental group and received one bone graft or served as control (cf. Table 1).

After the animal received their bone grafts (groups 2–4) or the empty circular bone defect (group 1), the flap was repositioned and the wound was sutured (5–0 Ethilon suture, Ethicon, Noderstedt, Germany). The rats received an antibiotic cover one time (amoxicillin-trihydrate, sc injection, 15 mg/kg bw, Fort Dodge Veterinär GmbH, Würselen, Germany) and a pain medication for four days (carprofen, sc injection, 4 mg/kg bw, Sigma-Aldrich, Darmstadt, Germany).

To assist the healing process the animals were fed a soft diet for the first three days and a regular diet afterwards. The behavior of the experimental animals was observed daily and the body weight was measured every two weeks.

At each time point for analysis the animals were sacrificed via carbon dioxide overdose.

#### Polyfluorochrome labeling

Fluorescent labels were incorporated in calcifying tissues and provide time marks of the growing bone [24]. The sequential intravital labeling of the regenerating bone was performed using the fluorochrome dyes Alizarine complexone (20 mg/kg bodyweight (bw)) and Calcein green (30 mg/kg bw) seven and three days prior to sacrifice, respectively.

### Multimodal imaging

#### Sample preparation for MRI

The animals were sacrificed after six, nine and twelve weeks of healing time. The cranium was dissected and promptly stored in a phosphate buffer solution containing penicillin, streptomycin, gentamycin and amphotericin B at 8°C.

#### MRI examination

The tissue samples were removed from the storing solution and cut to a suitable sample size. The specimens were immersed into a 20-mm-NMR-tube with a perfluorinated fluid (Fluorinert^™^ FC-77, 3M Belgium NV/SA, Zwijndrecht, Belgium) which prevents dehydration and contributes no proton signal during MRI measurements.

MR imaging was performed using a 7-T Bruker Avance nonclinical NMR spectrometer with a vertical-bore magnet (300 MHz Larmor frequency for protons, Bruker BioSpin, Rheinstetten, Germany) using a linear polarized birdcage radiofrequency coil of 20 mm inner diameter and a Bruker Micro 2.5 gradient system generating a maximum magnetic field gradient strength of up to 1 T/m on three axes. The experimental parameters used for imaging are summarized in Table 2.

**Table 2:** Summary of the chosen experimental parameters for MR imaging.

General imaging parameters			Coronal sections	
software		Paravision 5.0^1^	lateral resolution	0.04 x 0.08 mm
anatomical planes		coronal, axial	interslice distance	0.25 mm
matrix size		512 x 256 Px	number of slices	10
signal averages		32	slice thickness	0.20 mm
Contrasts			Axial sections	
PD^2^	T_R_^3^/T_E_^4^	3000/ 8 ms	lateral resolution	0.04 x 0.12 mm
T_1_	T_R_/T_E_	1200/ 8 ms	interslice distance	none
T_2_	T_R_/T_E_	5000/30 ms	number of slices	20

^1^Bruker BioSpin, Rheinstetten, Germany;

^2^PD… proton density;

^3^T_R_… repetition time;

^4^T_E_… echo time

#### Sample preparation for histology

The tissue samples were fixed in 4% formaldehyde immediately after the MRI examination. To extract the water from the tissues, the samples were treated in a graded series of ethanol. Then, all specimens were embedded in methylmethacrylate (Technovit^®^ 9100 NEU, Heraeus Kulzer, Wehrheim, Germany). Coronal sections were prepared according to Donath’s sawing and grinding technique [4]. Subsequently, the four central sections were polished (thin-section thickness ~ 30 μm) and stained with Masson-Goldner trichrome staining, allowing the distinct classification of mineralized tissue (green), non-mineralized osteoid (red-orange) and collagenous soft tissue (orange).

### Data analysis

#### Measured values

To demonstrate the practice of a concordance analysis the quantitative values of the newly formed bone within the artificial defect (bone volume, *BV*, [%]) and remaining defect width (*rDW*, [%]) have been assessed with both quantitative MRI and histomorphometry. The data measurements were realized by two examiners, one person for quantitative MRI (CE) and one for histomorphometry (MH).

#### Quantitative MRI

**Fig 2:**
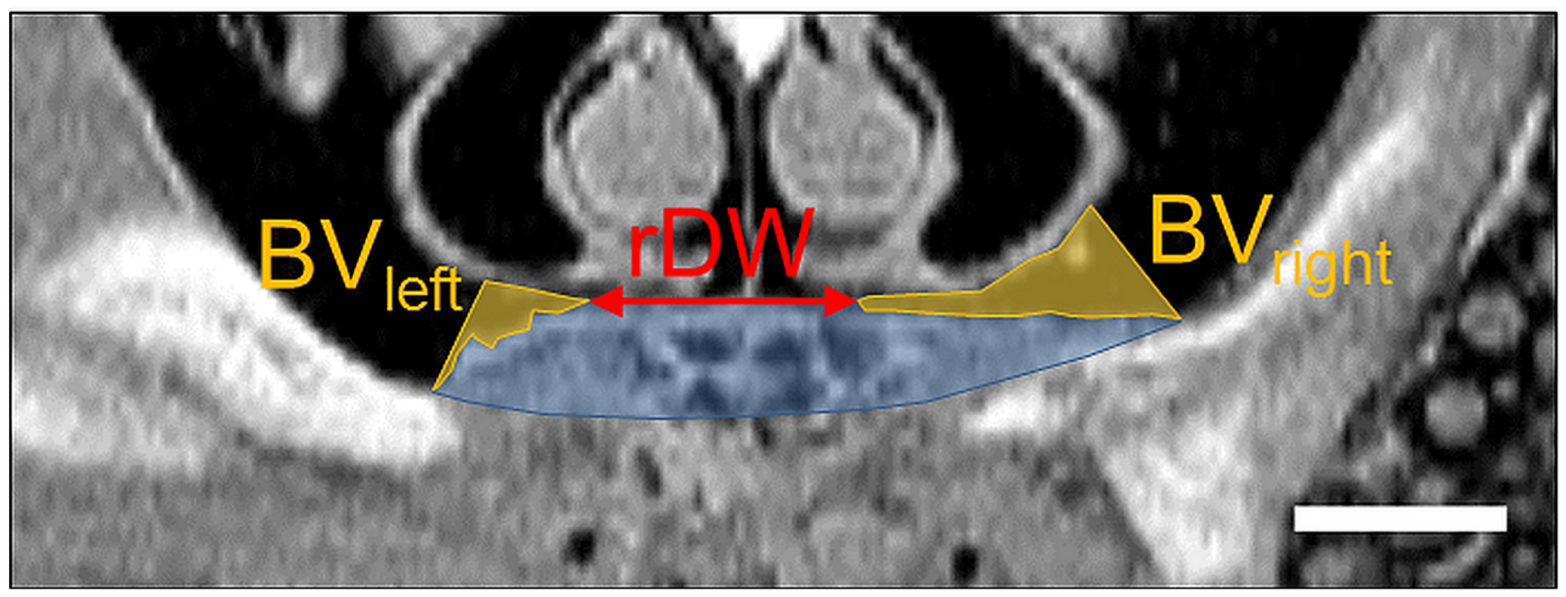
Measured parameters used for the concordance analysis. Here, a MRI slice image of a control was shown exemplarily. A detailed description of the tissue structures is given in Fig 3 and Table 3. To determine the overall newly formed bone (*BV*), the bone tissue area at the left and the right side of the artificial defect was measured. The remaining defect width (*rDW*) is marked with the red arrow. The scale bar represents 1.0 mm.

All datasets have been evaluated in Matlab^®^ version R2012a (Mathworks, Natick, USA). Quantitative MRI measurements were performed using MRI images with proton density contrast since there is the best signal-to-noise ratio. The four slice images that include the center of the artificial defect were chosen. The overall original defect area was measured together with the newly formed bone at the right and left side of the defect to obtain the percentage of newly formed bone (*BV*, [%]). Additionally, the remaining defect widths (*rDW*, [%]) were measured between the defect margins (Fig 2).

#### Histological analysis

All samples were imaged by fluorescence microscopy and, after staining, by light microscopy (Olympus BX 61, Olympus Deutschland GmbH, Hamburg, Germany). Multiple image alignment was performed using an automatic scanning table (Märzhäuser, Wetzlar, Germany). Thus, six images per sample were scanned with a 10x10 fold magnification and manually fused to one image working with the cell^F^®^ software (Olympus Soft Imaging Solutions GmbH, Münster, Germany). The histological analysis measured the same parameters like quantitative MRI.

#### Repeated measurements—Influence of the slice position

To zero out the influence of the chosen slice positions one single slice image was selected for evaluation exemplarily which depicts an equivalent layer with MRI and histology. The two quantitative values (*BV*, *rDW*) were measured 20 times for the single layer with both quantitative MRI and histomorphometry respectively and then compared.

#### Statistics

Statistical analysis was performed using Origin 9.1 (OriginLab Corporation, Northampton, USA) and SPSS Statistics 22 (IBM Cooperation, Armonk, USA).

To summarize the clinical success of the bone grafts investigated, the results were presented with bar graphs including the 95% confidence intervals of the mean values. The length of the 95% CI is calculated from the standard error of the bias and the critical t value of the t distribution:

$$95\;\%\;CI\;\left(d\right):\;\left[d-\frac{s_{D}}{\sqrt{n}}\cdot t_{\left(df,1-\alpha /2\right)};\;d+\frac{s_{D}}{\sqrt{n}}\cdot t_{\left(df,1-\alpha /2\right)}\right]$$

d… reference value, e.g. mean or bias, s_D_… standard deviation, n… number of measured values,

d*f = n-1*… degrees of freedom, α… level of significance = 0.05

The MRI and histological results of the chosen animals for concordance analysis were compared together with the histological findings of all animals investigated. To evaluate the differences between the four experimental groups, one-way ANOVA with the Scheffé post hoc test was used.

The assessment of the degree of agreement between the quantitative findings of both techniques relies upon the same animals (they were indicated with »*« in Table 1). The concordance analysis comprised X-Y-scatter plots and Bland-Altman diagrams. Here, the average of the measured values is plotted as X-coordinate against the difference between them as Y-coordinate. In addition, the bias has been displayed as solid horizontal line and furthermore, the 95% limits of agreement (i.e. 1.96 · *S*_*D*_) were included. The line of equality (deviation of the measured quantity is zero) has been highlighted as thick solid line as well. The decision, whether the quantitative data are comparable or differ significantly, could be easily taken by including the 95% confidence intervals for the mean difference between the two methods of measurement [13].

Additionally, all values were analyzed by a linear mixed model including a fixed effect of the intersample variability for the measurement result. The normal distribution of the values was assumed.

By means of the repeated quantification, the influence of the selected slice position has been diminished. For this purpose, the bias including the 95% CI was calculated. Additionally, a conventional t-test for testing agreement between the two data sets was used.

## Results

### Clinical results

Four laboratory animals did not complete the study which leads to a survival rate of 95.2%. Three animals did not awake from the anesthesia and one rat died during the healing period. The remaining 80 animals showed uneventful wound healing. The artificial defects were covered by oral mucosa.

### Morphological evaluation with MRI and histology

#### Detectable tissue structures

**Fig 3:**
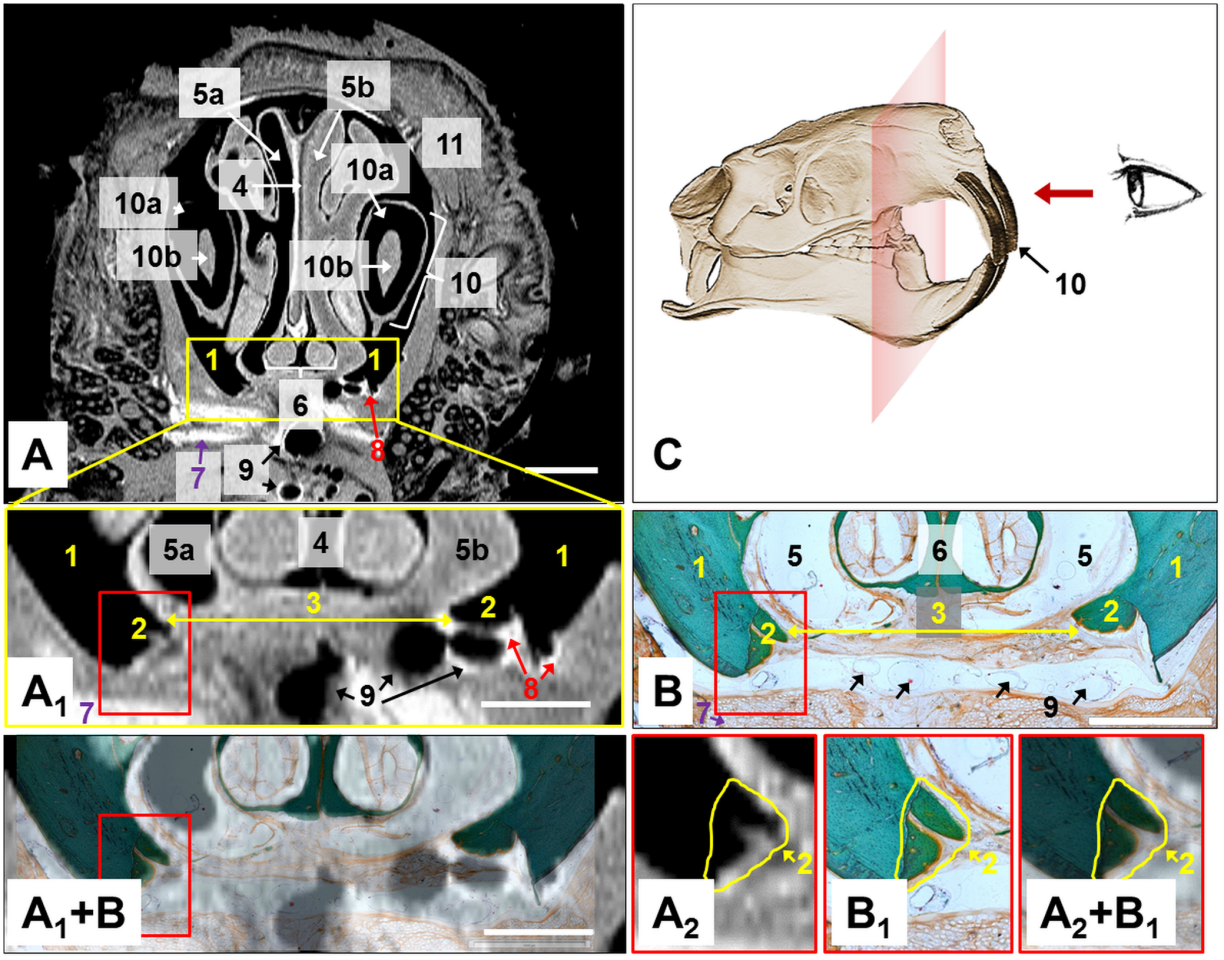
Coronal section of the maxilla and the skull with MRI and histology. The tissue structures are labeled and summarized in Table 3. One tissue sample of an animal from the control group is shown, exemplarily. The scale bars represent 1.0 mm. (A_1_) MRI, picture detail of the artificial defect. This view was used for image analysis. (A_2_) MRI, magnified view of the newly formed bone at the margins of the defect. (B) Histological image, Masson-Goldner trichrome stain. (B_1_) Magnified view of the newly formed bone. (A_1_+B) & (A_2_+B_1_) Superimposed MRI slice images with histological images. (C) Sketch of the front viewing direction of the coronal slices.

Both MRI and histology images showed detailed anatomical structures of the maxilla and skull. They were depicted and labeled in Fig 3 and Table 3.

**Table 3:** Detectable anatomical tissue structures of coronal slice images [26,27].

No.	Tissue structure
1	mature local bone (Os maxillare)
2	newly formed bone within the artificial defect
3	artificial defect, unfilled (control)
4	nasal septum
5	nasal cavity
5a	nasal cavity, filled with air
5b	nasal cavity, filled with soft tissue (mucosal swelling)
6	vomeronasal organ (Jacobson’s organ)
7	adipose tissue
8	susceptibility artifact
9	suture material or inclusion of air
10	incisor
10a	dental enamel and dentin
10b	dental pulp
11	soft tissue (skin)

In Fig 3, one exemplarily MRI slice image and the corresponding histological microscopy were compared. The entire maxilla and skull can be seen in the coronal MRI slice image (front view, cf. Fig 3A & 3C). The mineralized bone (No. 1, 2), dental enamel and dentin (No. 10a) appeared black (no MR signal). The adjacent soft tissue (No. 7, 11) and the fibrous tissue in the defect (No. 3) were imaged in different grey scales. In cranial direction, the nasal structures followed directly including the nasal septum (No. 4), nasal cavity (No. 5) and the vomeronasal organ (No. 6). The two incisors of the laboratory rat (No. 10) were conspicuously apparent on the MR slice image.

Newly formed bone could be identified directly at the margins with the help of the bone morphology and the visible bone structure upon the stained histological specimens (Fig 3B, No. 2) because of its disordered »woven« bone microstructure [25] and a different staining behavior in the case of the Masson-Goldner trichrome staining. Furthermore, the new bone created a cone-like structure and could also be distinguished from adult bone that way.

In the case of MRI images, the differentiation between mature and woven bone by means of different grey scales is not possible, but the newly formed bone could also be identified and quantified via bone morphology (cone-like new bone formation). Both imaging techniques clearly showed the wide artificial defect that was filled with fibrous tissue (No. 3) in the control group. Additionally, the suture material could be detected as almost colorless circles in the histological images (No. 9) and as black bubble-like structures (No. 9) on MRI images. Occasionally, these structures were superimposed by so-called susceptibility artifacts which preferentially occurred at interfaces in between the tissue incorporated air and the surrounding soft tissue in MRI images. These artifacts originate from local distortions of the static magnetic field due to materials properties (magnetic susceptibility) and are characterized by high signal intensities which appear in brighter greyscales compared to the surrounding soft tissues [28].

#### Image contrasts with MRI and histology

**Fig 4:**
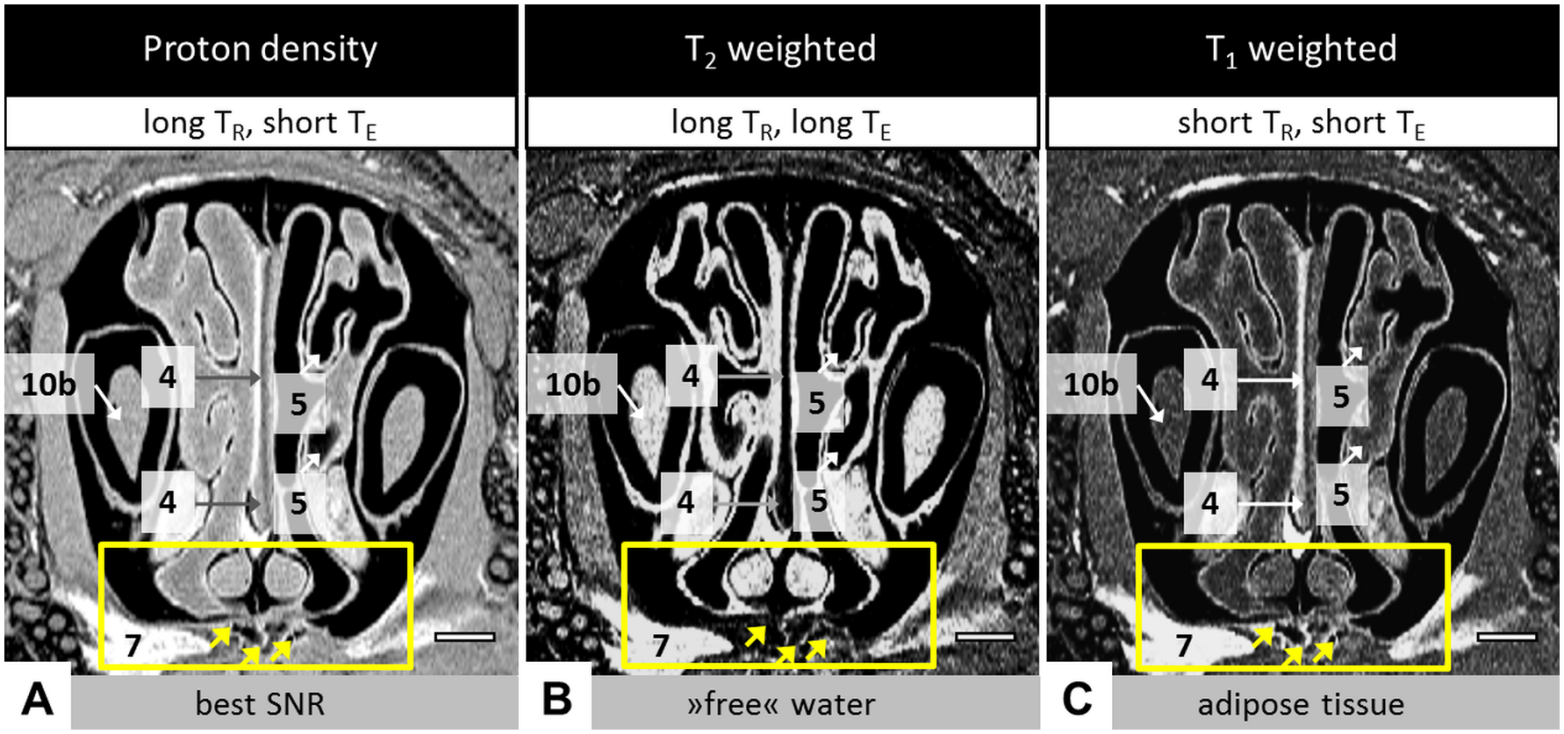
MRI images of the skull with different contrasts. Exemplarily, a specimen of group 2 after six weeks healing time was chosen. The yellow rectangle highlights the region of interest which includes the artificial defect. The arrows indicate the bone substitute material. The scale bars represent 1.0 mm. The repetition time and echo time which have been used for MR imaging (cf. Table 2) were abbreviated with T_R_ and T_E_, respectively. The label description can be also found in Table 3. (A) Proton density images which showed the best signal-to-noise ratio. They were used for quantitative MRI. (B) T_2_ weighted images highlight tissues with high content of unbound water. Adipose tissue appears bright, too. (C) With T_1_ weighted images the adipose tissue can be identified.

Contrast in MRI images is dominated by multiple factors: the signal intensity of an imaged tissue depends from the number of (mobile) protons within the tissue and the chosen imaging sequence, parameters and furthermore, the field strength which is determined by the MRI device. To evaluate the best imaging parameters for the cleft-like model, three different MRI contrasts were generated (Fig 4).

As expected, the proton density images showed the best signal-to-noise ratio (SNR) and were used for further quantification (Fig 4A). With so-called T_2_ weighted images the regions with a high content of unbound water could be identified (Fig 4B) [29]. They were found close to the mucosa of the nasal cavity (No. 5). Also high free water content was monitored in the dental pulp (No. 10b).

T_1_ weighted images are sensitive to adipose tissue and have been used for soft tissue differentiation in a previous study [30]. In the present case, adipose tissue could be identified in the vicinity of the nasal septum (No. 4) and as part of the connecting tissue in caudal direction to the artificial defect (No. 7).

The bone substitute material was visible as a granular structure. It appeared as particle clusters of different sizes and was enclosed by soft tissue. Using proton density contrast images allowed the best visibility of the material (cf. Fig 4A).

On the other hand, the character of histological images is determined by the chosen staining procedure. Various dyes are available to create the desired tissue contrast, whereas the Masson-Goldner trichrome staining is the golden standard to evaluate undecalcified plastic embedded bone [31]. Polychrome sequential labeling enables a second image representation for monitoring dynamics in bone growth and remodeling [24]. Accordingly, two image contrasts per histological tissue section are available.

**Fig 5:**
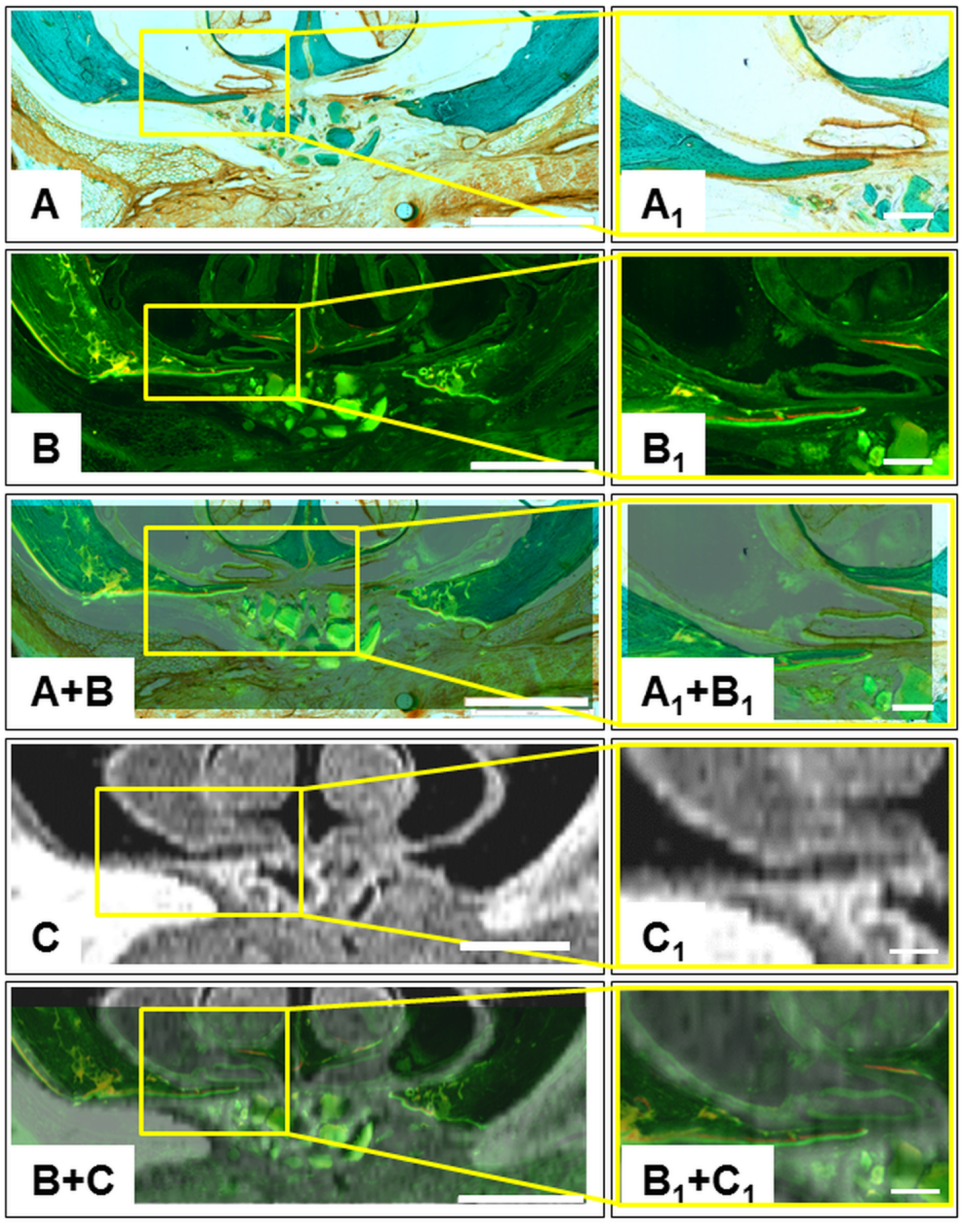
Multimodal representation of the artificial defect area. The same specimen of group 2 was chosen as displayed in Fig 4. The right column shows image magnifications of the newly formed bone tips. The scale bars represent 1.0 mm and 0.2 mm, respectively. (A & A_1_) Stained histologic sections. (B & B_1_) Fluorescence micrographs, the sequential polyfluorochrome labeling with Alizarine red and Calcein green highlighted the newly formed woven bone. (C & C_1_) Corresponding MRI images of the defect area. (A+B, A_1_+B_1_, B+C, B_1_+C_1_) If the stained sections or MRI images are superimposed with the Fluorescence micrographs, the recent mineralized bone tissue is clearly visible.

Particularly, the recent mineralized bone could clearly be identified in the stained microscopic images and the fluorescence micrographs. By means of Calcein green the mineralized bone that was formed at the time of application was labeled sufficiently. Alizarin red was incorporated earlier and detectable as narrow fluorescence bands (Fig 5B_1_). Hence, polyfluorochrome labeling indicated that bone formation was originated from mature bone. This became quite clear by superimposing the fluorescence micrographs with microscopic images of the stained sections (Fig 5A+B) and with MRI images (Fig 5B+C).

### Quantitative description of the defect healing

**Fig 6:**
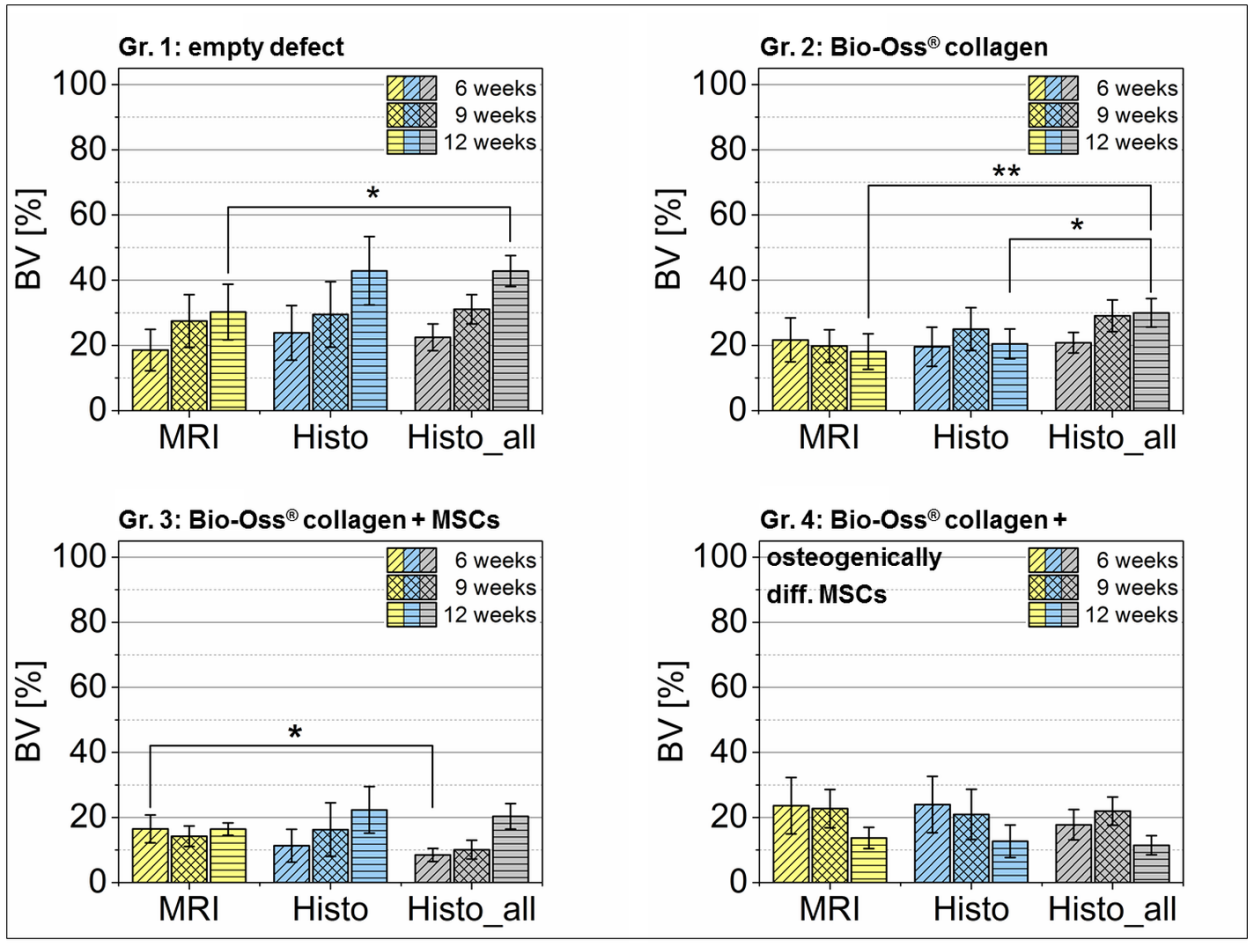
Quantitative results of the bone volume (BV) within the artificial defect. For comparison, the results of quantitative MRI and histomorphometry of the selected animals for method comparison (cf. Table 1) were presented with the measured *BV* value of all animals investigated in the study of *Korn et al*. [14]. The results were displayed as mean ± 95% CI. Statistical significance is indicated by *p< 0.05 and **p<0.01.

**Fig 7:**
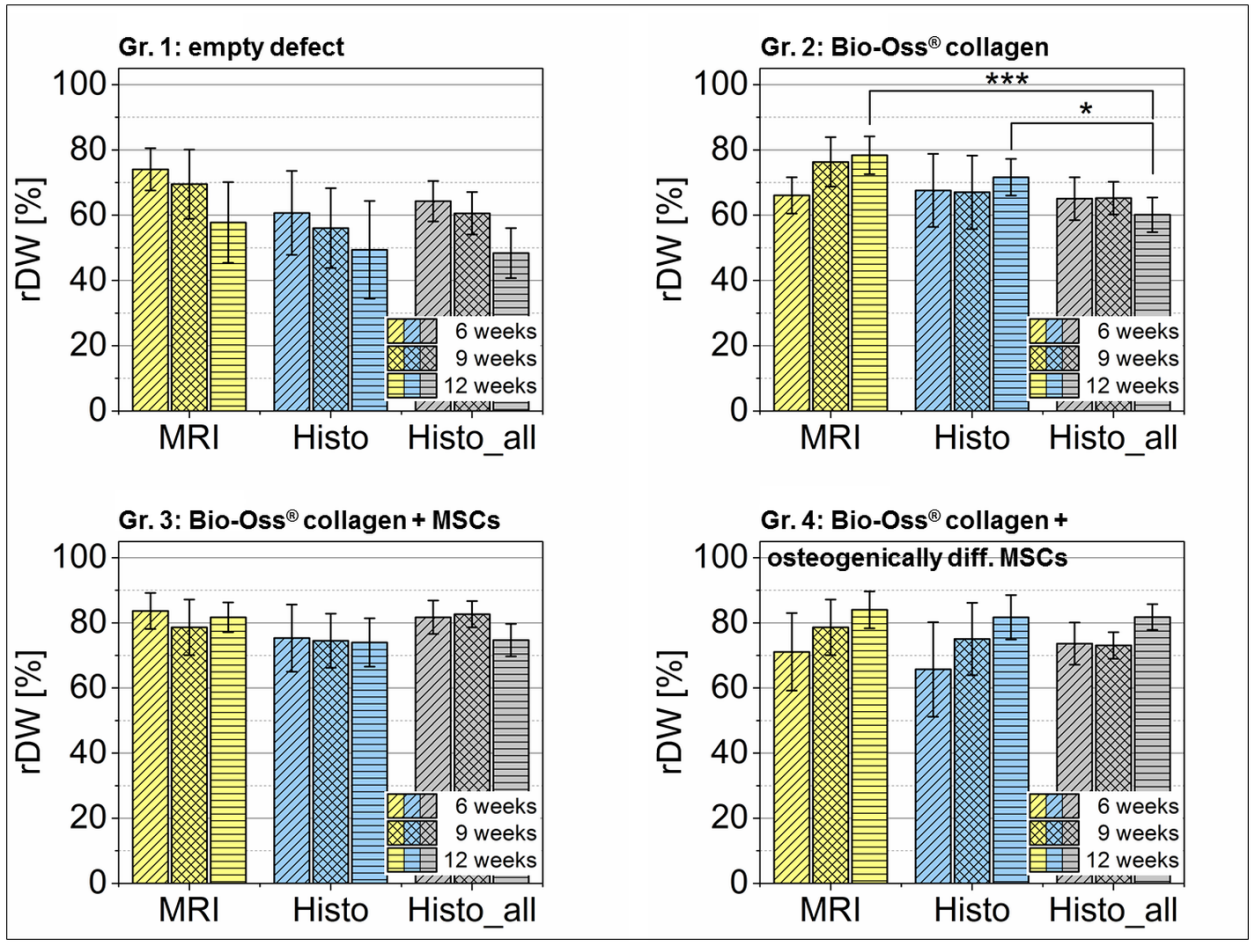
Quantitative results of the remaining defect width (rDW). In analogy to Fig 6, the results of quantitative MRI and histomorphometry of the selected animals for method comparison (cf. Table 1) were presented comparing the measured *rDW* value of all animals investigated in the study of *Korn et al*. [14]. The results were displayed as mean ± 95% CI. Statistical significance is indicated by *p< 0.05 and ***p<0.001.

In preparation for concordance analysis the bone formation within the defect area and the width of the defect margins have been quantified. The measured values of the bone volume within the defect (*BV*) and remaining defect width (*rDW*) were summarized in Figs 6 and 7 (cf. S1 Appendix: *Clinical results—Additional MRI and histological findings* and S2 Appendix: *Descriptive statistics*).

The labels *MRI* and *Histo* describe the amount of newly formed bone (BV, Fig 6) and the remaining defect width (rDW, Fig 7) that was received from the animals which were used for the agreement analysis only. On the whole, 28 laboratory animals have been examined in parallel (cf. Table 1). For comparison, the histological results of the overall group (cf. Table 1 and indicated as Histo_all) are presented in Figs 6 and 7, too.

The results were given as mean including the 95% confidence interval (CI) of the mean. This representation easily permits to draw conclusions to statistical significant differences. They can be assumed if the 95% CIs do not or overlay only marginally [32]. Due to the limited number of animals in the experimental groups that have been used for parallel MRI and histology, the Figs 6 and 7 could only give a first impression of the clinical results and the differences in the evaluation of the measured values. They are not suitable for assessing the degree of agreement between MRI and histology.

The results clearly demonstrated that the defect ossification was the most successful in group 1—which was the control group. Here, a continuous increase of newly formed woven bone has been quantified. After 12 weeks, the artificial defect was filled with mineralized tissue to a level of approximately 43% (*Histo_all*), meaning no complete bony reunion was observable. The same result can be seen with the remaining defect width (*rDW*). This parameter constantly only decreased in the control group.

The application of the bone graft according to the experimental design irrespectively whether it was tissue engineered or not, did not enhance the osteogenesis. Particularly in group 4 (BioOss^®^ collagen, with osteogenically differentiated MSCs) a significant decrease of the bone volume between weeks 9 and 12 occurred which was identifiable by histomorphometry as well as quantitative MRI.

### Assessing agreement using Bland-Altman analysis

**Fig 8:**
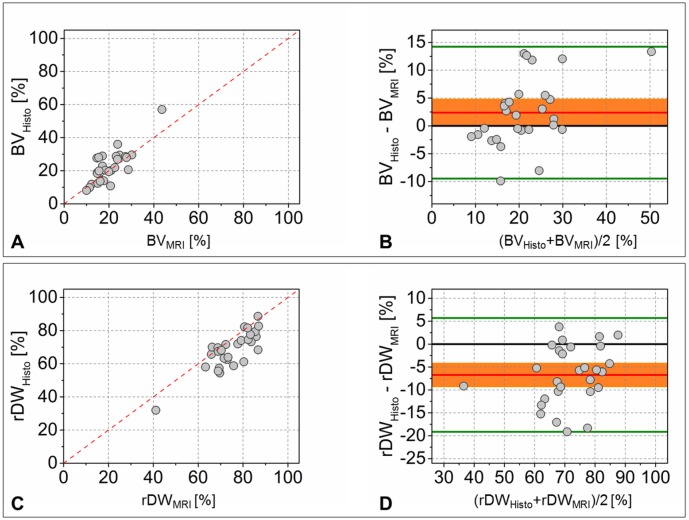
Concordance analysis for the measured values BV. (A, B) and *rDW* (C, D). X-Y-scatter plots are depicted (A, C) together with their corresponding Bland-Altman diagrams (B, D). --- angle bisector| ─ equality line| ─ mean difference (bias)| ▬ 95% CI for the mean difference| ─ limits of agreement, ± 1.96·s_D_.

To measure agreement between the two analyzing techniques investigated in the present study, quantitative MRI and histomorphometry, the evaluation of the bias between the mean differences is needed. A so-called concordance analysis includes X-Y-scatter plots together with Bland-Altman diagrams [12]. The X-Y-scatter plot gives a first impression of the overall measurement data and displays a potentially trend to one or the other method. The use of Bland-Altman diagrams allows a clear representation of the mean difference between two methods of measurement. Including the 95% CI of the bias the magnitude of the systematic deviation becomes evident. If the line of equality is not within the interval, the mean difference will be significant, i.e. the two methods quantified different values. In Fig 8 the graphical concordance analysis for the quantified bone volume (*BV*) and remaining defect width (*rDW*) is represented.

In the case of *BV* the X-Y-scatter plot (Fig 8A) displays a slight shift to larger histomorphometrical values. In the corresponding Bland-Altman diagram (Fig 8B) the bias is clearly visible. Whereas five values obtained from the histological images differed more than 10% from the corresponding MRI value, only one MRI result measured 10% higher than the corresponding histomorphometrical quantity. Taken as a whole, the mean difference of *BV* values was calculated to 2.4%. That means the histomorphometrical result was on average 2.4% higher than the MRI values for *BV*. The confidence interval of the mean difference has been calculated with [0.025 4.709]%, hence, the equality line was closely not included and a significant bias has been identified. Using the alternative mixed model analysis to calculate the probability value for comparison of the measurement methods (*p* = 0.010) the results from MRI quantification and histomorphometry differed clearly significantly.

In the case of the measured remaining defect width the result of the statistical comparison was clear without ambiguity. By means of the X-Y-scatter plot a considerable trend to higher MRI results was detectable, because the majority of the *rDW* values were located beneath the angle bisector. In the Bland-Altman diagram a wide distance of the equality line from the 95% CI of the mean difference was found (95% *CI* = [−9.186 − 4.271;*bias* = −6.729%]), indicating clear significant differences between the quantified data. The graphical statistical analysis was clearly confirmed by the calculated probability value of the mixed model (*p* = 6.801 · 10^−9^).

#### Influence of the slice position

**Fig 9:**
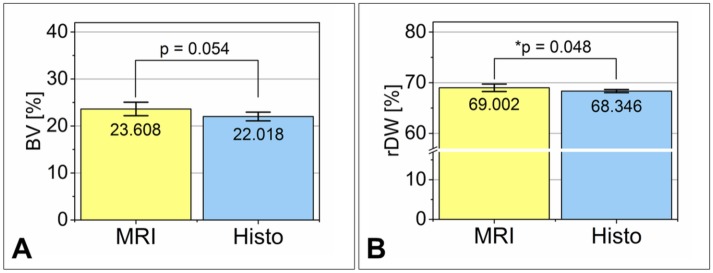
Depiction of the results from the repeated measurements (n = 20). On the whole, the quantified values from both data sets are close together. The findings of the t-test confirm the statements of Table 4.

The quantitative data acquisition for the study was performed by two examiners who selected the slices for quantification of *BV* and *rDW* independently of one another. For that reason, there might be a possible impact of the chosen slice position to the measured values. To eliminate the influence of the slice position one single section had been selected and evaluated in parallel for 20 times with MRI and histomorphometry. The results of the repeated quantification were summarized in Table 4 and Fig 9.

**Table 4:** Descriptive statistics of the repeated quantification (number of repetitions: 20).

Data set	Parameter	Mean	s_D_	Length, 95% CI	Lower 95% CI	Upper 95% CI
MRI	*BV* [%]	23.608	3.072	1.438	22.170	25.045
Histo	*BV* [%]	22.018	1.978	0.926	21.093	22.944
Histo-MRI	*BV*_Bias_ [%]	-1.589	3.450	1.615	-3.204	0.026
MRI	*rDW* [%]	69.002	1.574	0.736	68.266	69.739
Histo	*rDW* [%]	68.346	0.651	0.305	68.041	68.650
Histo-MRI	*rDW*_Bias_ [%]	-0.657	1.386	0.649	-1.305	-0.008

The results for quantitative MRI and histomorphometry are summarized together with their calculated deviation. In the case of *BV*, the 95% CI closely contains the line of equality (bias not significant). The deviation for *rDW* tightly excludes the zero-difference (significant bias).

The repeated evaluation of one single slice revealed a (not statistically significant) bias for *BV* of -1.6%, i.e. the MRI values were 1.6% larger on average (cf. Fig 9). Even though the results of both data sets were close together, this finding differed from the study result which was shown in the paragraph above. Including the measures of all available sections the average MRI value was 2.4% smaller on average than the histomorphometrical finding.

**Fig 10:**
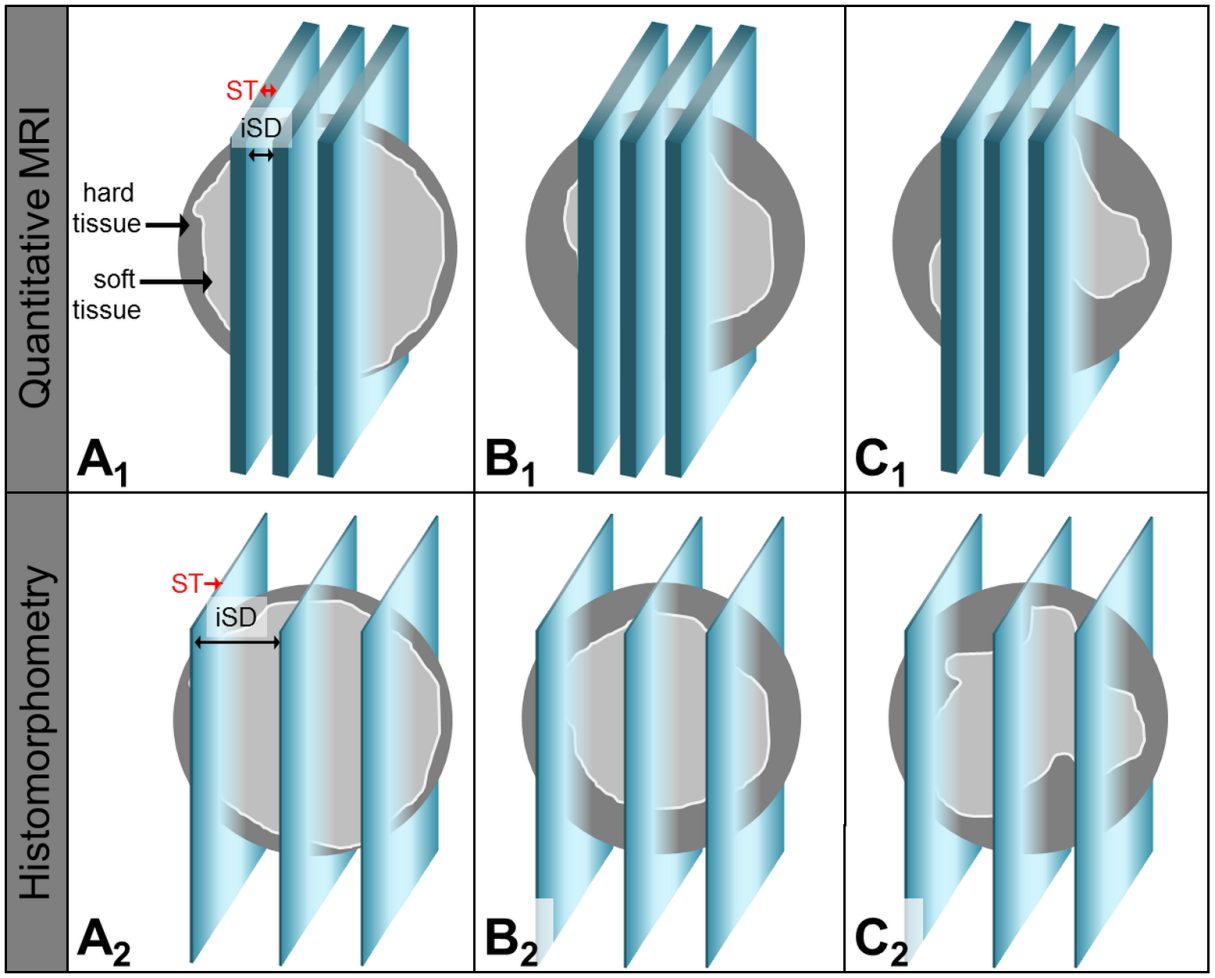
Sketch of the bone formation within the circular artificial cleft-like defect over time and the selected slice positions for quantitative MRI and histomorphometry. With increasing healing time (A… 6 weeks, B… 9 weeks, C… 12 weeks) the content of newly formed bone (*BV*) rises and grows from the defect margins to the center of the defect. *BV* analysis has been performed via quantitative MRI (upper row) and histomorphometry (lower row). The slices for MRI analysis were located centrally within the defect, showed a slice thickness (ST) of 200 μm and an interslice distance (iSD) of 250 μm. As a result of the histological preparation of hard tissue the sections had been very thin (ST ~ 30 μm), covered a wide range of the defect size (iSD ~ 1 mm) and also included regions close to the defect margins.

In case of *rDW* the mean difference was only 0.7%. Though the values were very close to each other the bias was tightly statistically significant. By comparing this result to the findings of the whole study, the mean difference of the repeated measurements had dropped nearly by 90%. This result clearly indicated that the examiners must have assessed different slice positions during the study. It is reasonable to assume that for MRI more central slice positions have been selected (Fig 10).

As a result of the histological sawing and grinding procedure large interslice distances occurred, thus, also sections were chosen for histomorphometry that had been located close to the defect margins. Because osteogenesis originated from the mature bone the sections adjacent to the defect margins showed a larger amount of newly formed bone compared to the center of the artificial defect. This well explains the on average larger histomorphometrical BV values compared to MRI where all available sections have been analyzed.

## Discussion

The vast majority of preclinical studies which monitor and evaluate the *in vivo* response of new materials use histological techniques [3]. However, for these methods the laboratory animals always have to be sacrificed at each analyzing time point. Subsequently, it is not possible to monitor the healing process repeatedly with one and the same animal which results in a large number required for a particular study and an increased biological variability. Research and establishment of non-invasive imaging techniques are therefore of essential importance in the field of biomaterials to comply the 3R policy (Replacement, Reduction and Refinement) [33].

Very detailed and recommended articles have been published that describe preclinical imaging technologies[2, 6, 34], but only a few show a real agreement analysis for different imaging modalities. The literature research revealed that there is no consensus in the biomaterial field and also in medicine how to compare different methods of measurement properly [35]. Most of the studies included correlation diagrams for comparisons (e.g. *Particelli et al*. [36]; *Kirschner et al*. [37]), some showed Bland-Altman plots additionally (*Bernhardt et al*. [38]; *Ferrare et al*. [39]). Interestingly, method comparisons between MRI and a second imaging technique are less common.

Therefore, the objective of the present study was to display a contrasting juxtaposition of the image qualities gained with MRI and histology, and, additionally, to show how an agreement analysis could be easily performed.

The basis of the present research article was the independent preclinical study of *Korn et al*. [14] which was planned only with an histological assessment originally. The research group has kindly given the allowance to use some of the (*ex vivo*) tissue samples for MRI analysis which enabled to perform the study. In doing so, the number of laboratory animals was kept as low as possible. The preclinical results and the findings of the concordance analysis were now briefly summarized in the next sections.

The preclinical study investigated the application of tissue-engineered bone grafts that have been used in an artificial, cleft-like maxillary defect. As scaffold material the clinically established bone graft Bio-Oss^®^ collagen was applied. Four experimental groups have been analyzed, one group was treated with the unmodified bone graft, two of the four groups received the tissue-engineered material and one group remained untreated and served as control (cf. Table 1). The study undeniably showed that treating the cleft-like artificial defect with the chosen bone grafts, irrespectively of whether tissue engineered or not, did not enhance the osteogenesis.

The main cause was probably the use of a granular scaffold material. The histological micrographs and MRI images revealed that the biomaterials did not remain within the artificial defect, but the bone grafts were dislocated to the adjacent tissue due to the masticatory forces (cf. S1 Appendix: *Clinical results—Additional MRI and histological findings*). It is likewise conceivable that the biomaterials were lost during ingestion, since individual sections of animals that received any bone graft showed nearly empty defect areas. The general result of the preclinical study was that the effect of the tissue engineering approach to healing process cannot be evaluated because the selected scaffold material turned out to be inappropriate.

Further preclinical studies should investigate scaffolds that are mechanically stable to withstand the masticatory forces during ingestion. Since a cleft palate is a large critical size defect that is influenced by strong masticatory forces [40], a stable and permanent bone substitute is essential that first enables a direct contact to mature bone and second promotes the formation of new bone produced naturally in the patient [18]. That is why the tissue engineering approach is very promising, even if the present study has shown a negative clinical result.

The bony ingrowth of the selected bone grafts and the formation of new mineralized tissue have been investigated with conventional histological techniques and MRI as an alternative imaging modality. First, the detectable anatomical structures of the artificial cleft-like defect were presented. Both imaging methods enabled a detailed impression of the maxilla and the skull. With the histological Masson-Goldner trichrome staining the newly formed woven bone was clearly distinguishable from the mature bone; whereupon the application of polyfluorochrome dyes identified that the new bone formation originated from the defect margins. The great advantage of MRI is its possibility to generate different image contrasts by means of varying experimental parameters. These can be used to differ between and even to quantify different tissue types [30]. In the present study, the use of so-called proton density images showed the best image qualities.

Additionally to the qualitative description of the defect healing, quantitative parameters were measured by means of both the histological micrographs and MRI images. The quantified values of the bone volume (*BV*, [%]) within the defect and the remaining defect width (*rDW*, [%]) were used to evaluate the agreement between the quantitative results. For that purpose, a graphical concordance analysis including X-Y-scatter plots and Bland-Altman diagrams was performed and validated using a linear mixed model analysis, supplementary. A fast and clear depiction of statistically relevant measurement deviations was achieved using the approach of *Giavarina* who recommended the inclusion of the 95% CI of the bias in the Bland-Altman diagram [13]. In doing so, the measurement bias can be easily read from the Bland-Altman plot, because it is possible to say that the bias is significant in the case the line of equaliy (showing zero difference) is not within the 95% CI of the mean difference. He also mentioned that this would be the analytical way and it could be necessary to define the agreement intervall more narrow for biological or clinical goals. But to the best knowledge of the authors, in the case of preclinical or clinical imaging no characteristic value for a desired agreement exists. Therefore, the analytical approach seems to be the best approach.

The comparison of the *BV* values identified an average bias (Histo-MRI) of 2.4% (*p* < 0.05)

, i.e. the histomorphometrical values were slightly but significantly larger than the BV values obtained from MRI images. In the case of *rDW* the concordance analysis revealed a distinct and significant difference (Histo-MRI) of -6.7% (*p* ≪ 0.05).

The cause of this could be discovered by repeating the measurements with one individual slice. In doing so, the impact of the slice position for the quantified results became clear. The study showed that for analyzing the same slice positions the quantified *BV* values were comparably

$$\left(Bias_{Histo-MRI:BV}=1.6\;\%,\;p=0.054\right).$$

The measured remaining defect width *rDW* differed only slightly

$$\left(Bias_{Histo-MRI:rDW}=-0.7\;\%,\;p=0.048\right).$$

Consequently, the calculated bias of the MRI and histomorphometrical data sets are less originated due to the imaging modalities themselves, but mainly on the evaluation of different slice positions. It can be assumed that for MRI the more central section were analyzed, while the histomorphometrical assessment used slice positions that were located closer to the defect margins. When considering that the osteogenesis originated from the mature bone, the identified bias of larger histomorphometrical *BV* and smaller *rDW* values becomes comprehensible.

The simplest way to reduce the average deviation would be to ensure that the examiners analyze really the same sections or to choose only one examiner who interprets all the imaging data alone. But, would it be honestly? And moreover, the data evaluation with several persons corresponds to the real situation in the case of interdisciplinary projects.

Now then, the result of the present study still leaves open the question, whether few two-dimensional section really represent the whole three-dimensional bone architecture [2,5]. Practitioners of histological techniques have to be aware that although the lateral resolution of the micrographs from histology is excellent the resolution in longitudinal direction is poor (cf. Fig 10). Due to the extensive and destructive histological sample preparation only a few (usually three to five) sections are obtained which have a distance from each other. The histomorphometrical results are always extrapolated to the situation in the whole volume that comes along with a measurement error. The extent is difficult to estimate and will be the larger the more non-uniform the distribution of different tissues is. E.g. if the new bone formation around a dental implant is nearly completed, an isotropic osseous situation will be found. In this case the evaluation of three to four microscopic sections is proven to be sufficient [38]. But bone formation during the early healing stages cannot be assumed to be uniform [30].

To describe the overall volume of the defect area more exactly, the analysis of a larger amount of slices or a three-dimensional data evaluation is needed. Consequently, the investigation of alternative imaging techniques for various kinds of preclinical models is of crucial interest.

## Conclusions

The use of animal models in preclinical biomaterial research represents a bridge between the findings of basic sciences and their clinical implementation. To evaluate new materials and engineered tissues, the use of non-destructive imaging modalities have been identified as a strategic priority. This provides a unique opportunity for studying the interactions between biomaterial and host tissue repeatedly with individual animals, along with the advantage of a reduced biological variability, decreased number of required laboratory animals and the possibility of three-dimensional data evaluation. However, histological techniques are still the golden standard in preclinical research projects. For establishing alternative imaging modalities to investigate novel biomaterials, the direct comparison of the image qualities and quantitative results to histology is therefore indispensable.

Although the experimental effort using parallel imaging will be larger, the statistical analysis whether the findings of the different imaging techniques used for a particular study are comparable to each other or not, can be easily performed without certain statistic software. Within the present study it could be proven that by using a graphical concordance analysis including X-Y-scatter plots and the extended Bland-Altman diagrams the mean difference between MRI results and histomorphometrical measures was clearly identifiable. The graphical findings were also in accordance with the statistical mixed model analysis.

Moreover, the article showed that method comparisons not always need the use of an independent animal study because at many research centers various different preclinical projects have already been performed. Such interdisciplinary projects offer the opportunity to answer several scientific questions with one and the same experiment and help to reduce the number of laboratory animals.

## Supporting information

S1 Appendix Clinical results—Additional MRI and histological findings. (PDF)

S2 Appendix Descriptive statistics. (PDF)

S3 Appendix Bland-Altman analysis and plot. (PDF)

S4 Appendix Repeated measurements—Influence of the slice position. (PDF)
