# Supplementary material for: Assessing agreement between preclinical magnetic resonance imaging and histology: An evaluation of their image qualities and quantitative results
Source: PLoS One. 2017 Jun 30;12(6):e0179249. doi: 10.1371/journal.pone.0179249 (PMC5493293; doi:10.1371/journal.pone.0179249)
Supplement: S1 Appendix — (PDF) [file pone.0179249.s001.pdf]

S1 Appendix: Clinical results – Additional MRI and histological findings

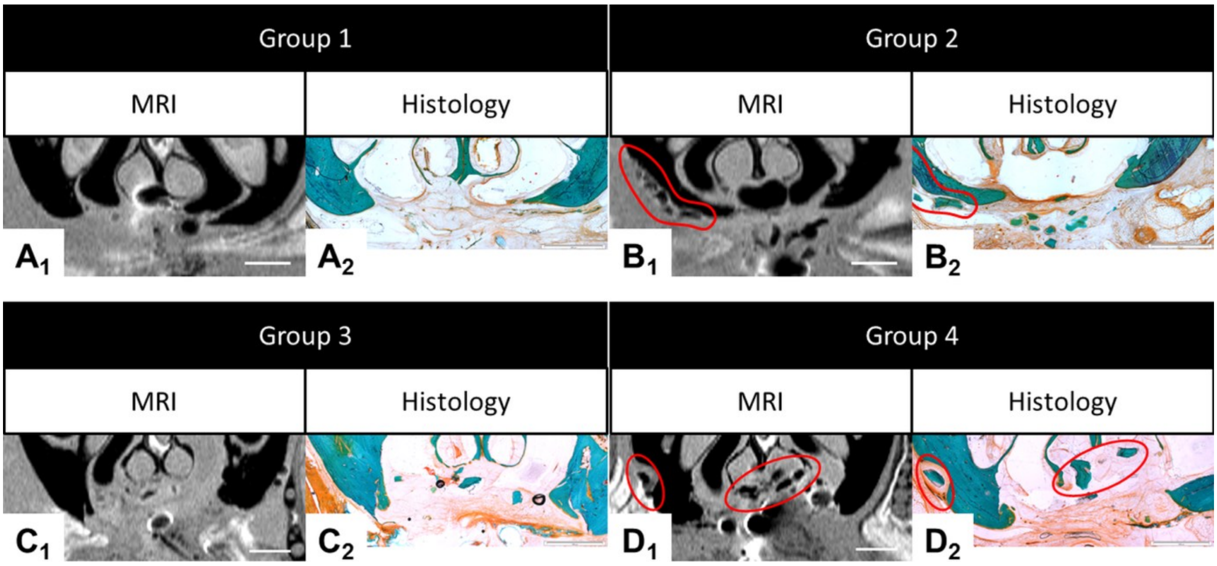

S1 Fig. 1 Exemplarily selected findings that demonstrate lost or dislocated bone grafts.
The images show the osseous situation after nine weeks healing time. Dislocated particles have been distinguished within the red line. The scale bars represent 1.0 mm. (A) Control group: wide, empty defect. (B) Bio-Oss® collagen: several particles of the bone graft have been dislocated to the caudal maxilla and induced bone resorption. (C) Bio-Oss® collagen, with MSCs: the bone substitute was lost during the healing time. (D) Bio-Oss® collagen, with osteogenic differentiated MSCs: highly dislocated particles that could be found within the Jacobson's organ and close to the caudal maxilla. The particles induced significantly bone resorption.

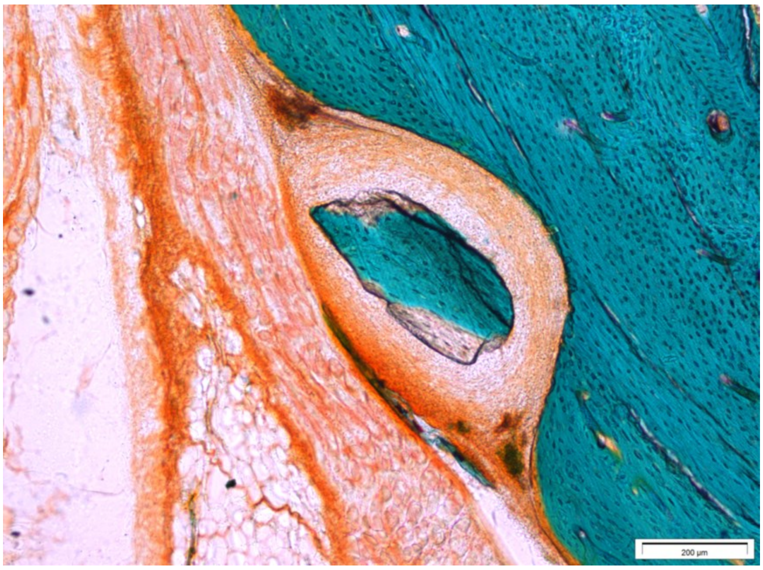

S1 Fig. 2 Isolated particle (cf. S1 Fig. 1D$_2$).
The dislocated bone graft have been encapsulated with fibrous tissue.
