# Supplementary material for: Assessing agreement between preclinical magnetic resonance imaging and histology: An evaluation of their image qualities and quantitative results
Source: PLoS One. 2017 Jun 30;12(6):e0179249. doi: 10.1371/journal.pone.0179249 (PMC5493293; doi:10.1371/journal.pone.0179249)
Supplement: S2 Appendix — (PDF) [file pone.0179249.s002.pdf]

S2 Appendix: Descriptive statistics

Results from quantitative MRI

S2 Table 1 Measured newly formed bone (BV), descriptive analysis, MRI quantification.

Group	Time [wk]	Parameter	N	d [%]	s_D [%]	SEM [%]	95 % CI [%]	d − 95 % CI [%]	d + 95 % CI [%]	Min [%]	Max [%]	Range [%]
1	6	BV	8	18,556	7,593	2,685	6,348	12,208	24,904	11,395	30,684	19,289
2	6	BV	8	21,688	8,062	2,850	6,740	14,948	28,428	11,491	31,798	20,307
3	6	BV	8	16,493	5,117	1,809	4,278	12,215	20,770	11,459	24,765	13,306
4	6	BV	8	23,626	10,386	3,672	8,683	14,943	32,309	12,086	42,793	30,707
1	9	BV	8	27,461	9,662	3,416	8,077	19,384	35,539	15,900	48,337	32,437
2	9	BV	8	19,818	5,936	2,099	4,963	14,855	24,781	13,083	31,701	18,618
3	9	BV	8	14,220	3,765	1,331	3,148	11,073	17,368	7,843	19,231	11,388
4	9	BV	8	22,751	7,034	2,487	5,880	16,871	28,631	12,008	31,530	19,522
1	12	BV	12	30,256	13,428	3,876	8,532	21,725	38,788	15,254	50,563	35,308
2	12	BV	12	18,108	8,613	2,486	5,472	12,635	23,580	7,918	32,080	24,162
3	12	BV	12	16,441	2,997	0,865	1,904	14,537	18,345	13,203	22,887	9,684
4	12	BV	12	13,702	5,127	1,480	3,258	10,444	16,960	6,591	20,453	13,862

d… mean value, N… number of MRI slices; s_D… standard deviation; SEM… standard error of the mean; CI… confidence interval

S2 Table 2 Measured remaining defect widths (*rDW*), descriptive analysis, MRI quantification.

Group	Time [wk]	Parameter	N	d [%]	s_D [%]	SEM [%]	95 % CI [%]	d − 95 % CI [%]	d + 95 % CI [%]	Min [%]	Max [%]	Range [%]
1	6	*rDW*	8	74,045	7,756	2,742	6,485	67,561	80,530	61,047	82,682	21,635
2	6	*rDW*	8	66,064	6,637	2,346	5,549	60,515	71,613	58,065	74,172	16,108
3	6	*rDW*	8	83,671	6,626	2,343	5,540	78,131	89,210	70,558	92,118	21,560
4	6	*rDW*	8	71,098	14,230	5,031	11,897	59,201	82,994	39,316	80,905	41,588
1	9	*rDW*	8	69,510	10,620	3,755	8,879	60,631	78,389	52,198	78,571	26,374
2	9	*rDW*	8	76,314	9,041	3,197	7,559	68,755	83,873	59,394	85,526	26,132
3	9	*rDW*	8	77,082	11,525	4,075	9,635	67,447	86,717	55,862	90,270	34,408
4	9	*rDW*	8	78,614	10,261	3,628	8,578	70,035	87,192	62,105	89,286	27,180
1	12	*rDW*	12	57,767	19,482	5,624	12,378	45,389	70,145	31,111	78,808	47,697
2	12	*rDW*	12	78,363	9,126	2,634	5,798	72,565	84,162	62,424	90,608	28,183
3	12	*rDW*	12	81,693	7,175	2,071	4,559	77,135	86,252	60,656	90,556	29,900
4	12	*rDW*	12	83,995	8,914	2,573	5,664	78,332	89,659	66,304	100,000	33,696

d… mean value, N… number of MRI slices; s_D… standard deviation; SEM… standard error of the mean; CI… confidence interval

Results from histomorphometry

S2 Table 3 Measured newly formed bone (*BV*), descriptive analysis, histomorphometry, animals of the whole study.

Group	Time [wk]	Parameter	N	d [%]	s_D [%]	SEM [%]	95 % CI [%]	d − 95 % CI [%]	d + 95 % CI [%]	Min [%]	Max [%]	Range [%]
1	6	*BV*	28	22,508	10,605	2,004	4,112	18,395	26,620	5,588	40,585	34,997
2	6	*BV*	32	20,842	8,690	1,536	3,133	17,709	23,975	0,556	42,905	42,349
3	6	*BV*	38	8,475	6,068	0,984	1,995	6,480	10,469	0,988	22,723	21,736
4	6	*BV*	30	17,748	12,471	2,277	4,657	13,092	22,405	1,526	45,149	43,623
1	9	*BV*	31	31,082	12,212	2,193	4,479	26,602	35,561	16,693	58,058	41,365
2	9	*BV*	29	29,093	12,799	2,377	4,868	24,225	33,962	13,956	69,329	55,374
3	9	*BV*	30	10,093	7,698	1,405	2,874	7,219	12,968	1,344	32,570	31,225
4	9	*BV*	31	21,968	11,800	2,119	4,328	17,640	26,296	4,151	63,157	59,006
1	12	*BV*	29	42,816	12,507	2,322	4,757	38,058	47,573	18,893	64,301	45,407
2	12	*BV*	33	29,997	12,279	2,137	4,354	25,643	34,350	11,553	56,328	44,774
3	12	*BV*	30	20,349	10,542	1,925	3,936	16,412	24,285	5,094	44,544	39,450
4	12	*BV*	26	11,463	7,276	1,427	2,939	8,524	14,402	1,878	34,856	32,978

d… mean value, N… number of histological sections; s_D… standard deviation; SEM… standard error of the mean; CI… confidence interval

S2 Table 4 Measured newly formed bone (*BV*), descriptive analysis, histomorphometry, animals that have been used for concordance analysis.

Group	Time [wk]	Parameter	N	d [%]	s_D [%]	SEM [%]	95 % CI [%]	d − 95 % CI [%]	d + 95 % CI [%]	Min [%]	Max [%]	Range [%]
1	6	*BV*	8	23,860	10,029	3,546	8,385	15,475	32,245	11,490	36,434	24,944
2	6	*BV*	9	19,575	7,781	2,594	5,981	13,594	25,556	11,630	33,979	22,349
3	6	*BV*	10	11,337	7,044	2,227	5,039	6,298	16,376	2,074	22,723	20,649
4	6	*BV*	10	23,999	12,127	3,835	8,675	15,324	32,674	3,605	45,149	41,544
1	9	*BV*	9	29,500	13,025	4,342	10,012	19,489	39,512	16,693	50,352	33,659
2	9	*BV*	9	24,999	8,545	2,848	6,568	18,431	31,567	13,956	41,721	27,766
3	9	*BV*	8	16,276	9,830	3,475	8,218	8,058	24,494	2,877	32,570	29,692
4	9	*BV*	8	20,953	9,213	3,257	7,702	13,250	28,655	8,903	37,753	28,850
1	12	*BV*	11	42,878	15,588	4,700	10,472	32,406	53,351	19,005	64,301	45,295
2	12	*BV*	12	20,493	7,193	2,077	4,570	15,923	25,063	11,553	33,076	21,522
3	12	*BV*	14	22,340	12,363	3,304	7,138	15,202	29,478	7,998	44,544	36,547
4	12	*BV*	13	12,703	8,255	2,289	4,988	7,714	17,691	4,680	34,856	30,176

d… mean value, N… number of histological sections; s_D… standard deviation; SEM… standard error of the mean; CI… confidence interval

S2 Table 5 Measured remaining defect widths (*rDW*), descriptive analysis, histomorphometry, animals of the whole study.

Group	Time [wk]	Parameter	N	d [%]	s_D [%]	SEM [%]	95 % CI [%]	d − 95 % CI [%]	d + 95 % CI [%]	Min [%]	Max [%]	Range [%]
1	6	*rDW*	26	64,283	15,403	3,021	6,221	58,061	70,504	34,738	85,887	51,149
2	6	*rDW*	30	65,053	17,553	3,205	6,554	58,499	71,608	21,370	90,713	69,343
3	6	*rDW*	35	81,722	14,928	2,523	5,128	76,595	86,850	43,070	99,767	56,697
4	6	*rDW*	30	73,628	17,328	3,164	6,470	67,158	80,099	38,441	96,791	58,350
1	9	*rDW*	29	60,582	17,136	3,182	6,518	54,064	67,100	21,475	83,628	62,154
2	9	*rDW*	26	65,244	12,431	2,438	5,021	60,223	70,265	32,606	85,290	52,684
3	9	*rDW*	28	82,660	10,473	1,979	4,061	78,599	86,721	64,705	98,296	33,591
4	9	*rDW*	30	73,071	9,582	1,749	3,578	69,493	76,649	54,876	90,502	35,626
1	12	*rDW*	27	48,396	19,416	3,737	7,681	40,715	56,077	9,451	94,423	84,972
2	12	*rDW*	31	60,135	14,489	2,602	5,315	54,821	65,450	24,310	82,943	58,633
3	12	*rDW*	28	74,697	12,865	2,431	4,989	69,708	79,685	52,190	91,886	39,696
4	12	*rDW*	26	81,784	9,740	1,910	3,934	77,850	85,718	56,668	91,082	34,413

d… mean value, N… number of histological sections; s_D… standard deviation; SEM… standard error of the mean; CI… confidence interval

S2 Table 6 Measured remaining defect widths (*rDW*), descriptive analysis, histomorphometry, animals that have been used for concordance analysis.

Group	Time [wk]	Parameter	N	d [%]	s_D [%]	SEM [%]	95 % CI [%]	d − 95 % CI [%]	d + 95 % CI [%]	Min [%]	Max [%]	Range [%]
1	6	*rDW*	8	60,700	15,379	5,437	12,857	47,842	73,557	34,738	80,435	45,698
2	6	*rDW*	9	67,598	14,586	4,862	11,212	56,386	78,809	40,027	82,859	42,832
3	6	*rDW*	10	75,321	14,401	4,554	10,302	65,018	85,623	43,613	88,596	44,982
4	6	*rDW*	10	65,706	20,281	6,413	14,508	51,198	80,214	38,441	93,101	54,660
1	9	*rDW*	9	56,063	15,972	5,324	12,277	43,786	68,340	26,687	72,382	45,696
2	9	*rDW*	9	67,007	14,655	4,885	11,265	55,742	78,271	32,606	85,290	52,684
3	9	*rDW*	8	74,498	9,941	3,515	8,310	66,187	82,808	64,705	94,602	29,897
4	9	*rDW*	8	75,037	13,278	4,695	11,101	63,936	86,138	45,954	86,932	40,979
1	12	*rDW*	12	49,386	23,578	6,807	14,981	34,405	64,367	12,378	94,423	82,045
2	12	*rDW*	12	71,638	8,871	2,561	5,636	66,002	77,274	59,161	82,943	23,782
3	12	*rDW*	14	73,975	12,856	3,436	7,423	66,552	81,398	52,738	91,529	38,791
4	12	*rDW*	13	81,714	11,311	3,137	6,835	74,879	88,549	56,668	90,783	34,114

d… mean value, N… number of histological sections; s_D… standard deviation; SEM… standard error of the mean; CI… confidence interval
