# Supplementary material for: Assessing agreement between preclinical magnetic resonance imaging and histology: An evaluation of their image qualities and quantitative results
Source: PLoS One. 2017 Jun 30;12(6):e0179249. doi: 10.1371/journal.pone.0179249 (PMC5493293; doi:10.1371/journal.pone.0179249)
Supplement: S3 Appendix — (PDF) [file pone.0179249.s003.pdf]

S3 Appendix: Bland-Altman analysis and plot

Bland-Altman analysis

Approximately four sections (either MR slice images or histological micrographs) have been used to quantify the parameters BV and rDW. For Bland-Altman analysis, the mean value per animal has been calculated and listed. Thus, each line shows paired data (columns 4 and 5). To evaluate the agreement of two methods the specific differences between one measurement (the MRI value) and the other (the corresponding histomorphometrical value) need to be studied for each pair (column 7). This is called the »bias«. If neither of the two methods is a »reference«, thus a true calibrated value, the differences were compared with the mean of the two paired values (column 6).

Ideally, all the differences, and consequently the mean difference $(\bar{d})$ (column 8), would be equal to zero (line of equality). In the case the differences were normally distributed, 95 % of the differences would lie between $\bar{d} - 1.96 \cdot s_D$ and $\bar{d} + 1.96 \cdot s_D$, ergo the two limits of agreement (columns 11 and 12). This is called the Bland-Altman plot system.

By now, it is not possible to say if the agreement is acceptable or if the bias is significantly. The analytical approach to analyze that is to calculate the 95 % confidence intervals (CI) for the mean difference: $\bar{d} - 95\ \%\ CI$ and $\bar{d} + 95\ \%\ CI$ (columns 9 and 10).

$$95\ \%\ \mathrm{CI}\ (\bar{d}):\ [\bar{d} - \frac{s_D}{\sqrt{n}} \cdot t_{(df,1-\alpha/2)}; \bar{d} + \frac{s_D}{\sqrt{n}} \cdot t_{(df,1-\alpha/2)}]$$

$\bar{d}$… mean difference = bias, s_D… standard deviation, n… number of measured values, $df=n-1$… degrees of freedom, α… level of significance = 0.05

In the case that the line of equality is not within the 95 % CI of the mean difference, the calculated bias is statistically significant.

S3 Table 1 Bland-Altman analysis for comparison of the measured newly formed bone (BV).

Animal No.	Time [wk]	Group	BV_{MRI} [%]	BV_{Histo} [%]	$\left(\frac{BV_{MRI}+BV_{Histo}}{2}\right)$ [%]	$BV_{Histo} - BV_{MRI}$ [%]	$\bar{d}_{BV}$ [%]	$\bar{d} + 95\,\%\,CI$ [%]	$\bar{d} - 95\,\%\,CI$ [%]	$\bar{d} + 1.96 s_D$ [%]	$\bar{d} - 1.96 s_D$ [%]
31	6	1	14,637	27,624	21,131	12,987	2,367	4,709	0,025	14,206	-9,472
32	6	1	20,907	20,097	20,502	-0,810	2,367	4,709	0,025	14,206	-9,472
2	9	1	30,205	29,554	29,880	-0,651	2,367	4,709	0,025	14,206	-9,472
3	9	1	24,717	29,458	27,088	4,741	2,367	4,709	0,025	14,206	-9,472
19	12	1	23,221	28,721	25,971	5,500	2,367	4,709	0,025	14,206	-9,472
20	12	1	43,654	56,983	50,319	13,329	2,367	4,709	0,025	14,206	-9,472
21	12	1	23,894	35,930	29,912	12,036	2,367	4,709	0,025	14,206	-9,472
40	6	2	14,765	18,784	16,775	4,019	2,367	4,709	0,025	14,206	-9,472
42	6	2	28,611	20,564	24,588	-8,047	2,367	4,709	0,025	14,206	-9,472
8	9	2	17,071	28,887	22,979	11,816	2,367	4,709	0,025	14,206	-9,472
9	9	2	22,565	21,889	22,227	-0,676	2,367	4,709	0,025	14,206	-9,472
22	12	2	15,717	18,392	17,055	2,675	2,367	4,709	0,025	14,206	-9,472
23	12	2	14,795	18,369	16,582	3,574	2,367	4,709	0,025	14,206	-9,472
24	12	2	23,812	26,834	25,323	3,022	2,367	4,709	0,025	14,206	-9,472
57	6	3	12,266	11,841	12,054	-0,425	2,367	4,709	0,025	14,206	-9,472
58	6	3	20,720	10,832	15,776	-9,888	2,367	4,709	0,025	14,206	-9,472

S3 Table 1, continued.

Animal No.	Time [wk]	Group	BV_{MRI} [%]	BV_{Histo} [%]	$\left(\frac{BV_{MRI}+BV_{Histo}}{2}\right)$ [%]	$BV_{Histo} - BV_{MRI}$ [%]	$\bar{d}_{BV}$ [%]	$\bar{d} + 95\,\%\,CI$ [%]	$\bar{d} - 95\,\%\,CI$ [%]	$\bar{d} + 1.96s_D$ [%]	$\bar{d} - 1.96s_D$ [%]
73	9	3	17,105	22,784	19,945	5,679	2,367	4,709	0,025	14,206	-9,472
74	9	3	11,335	9,768	10,552	-1,567	2,367	4,709	0,025	14,206	-9,472
45	12	3	15,392	28,036	21,714	12,644	2,367	4,709	0,025	14,206	-9,472
47	12	3	18,352	20,269	19,311	1,917	2,367	4,709	0,025	14,206	-9,472
48	12	3	15,579	19,855	17,717	4,276	2,367	4,709	0,025	14,206	-9,472
64	6	4	27,329	28,571	27,950	1,242	2,367	4,709	0,025	14,206	-9,472
65	6	4	19,922	19,427	19,675	-0,495	2,367	4,709	0,025	14,206	-9,472
80	9	4	27,818	27,956	27,887	0,138	2,367	4,709	0,025	14,206	-9,472
82	9	4	17,684	13,949	15,817	-3,735	2,367	4,709	0,025	14,206	-9,472
49	12	4	15,026	12,373	13,700	-2,653	2,367	4,709	0,025	14,206	-9,472
50	12	4	10,020	8,095	9,058	-1,925	2,367	4,709	0,025	14,206	-9,472
51	12	4	16,061	13,621	14,841	-2,440	2,367	4,709	0,025	14,206	-9,472

S3 Table 2 Bland-Altman analysis for comparison of the measured remaining defect widths (rDW).

Animal No.	Time [wk]	Group	rDW_{MRI} [%]	rDW_{Histo} [%]	$\left(\frac{rDW_{MRI}+rDW_{Histo}}{2}\right)$ [%]	$rDW_{Histo} - rDW_{MRI}$ [%]	$\bar{d}_{rDW}$ [%]	$\bar{d} + 95\,\%\,CI$ [%]	$\bar{d} - 95\,\%\,CI$ [%]	$\bar{d} + 1.96s_D$ [%]	$\bar{d} - 1.96s_D$ [%]
31	6	1	75,819	58,766	67,293	-17,053	-6,729	-4,271	-9,186	5,694	-19,151
32	6	1	72,981	62,633	67,807	-10,348	-6,729	-4,271	-9,186	5,694	-19,151
2	9	1	69,652	54,403	62,028	-15,249	-6,729	-4,271	-9,186	5,694	-19,151
3	9	1	69,368	57,391	63,380	-11,977	-6,729	-4,271	-9,186	5,694	-19,151
19	12	1	68,901	55,579	62,240	-13,322	-6,729	-4,271	-9,186	5,694	-19,151
20	12	1	41,115	31,951	36,533	-9,164	-6,729	-4,271	-9,186	5,694	-19,151
21	12	1	63,283	58,071	60,677	-5,212	-6,729	-4,271	-9,186	5,694	-19,151
40	6	2	65,879	65,660	65,770	-0,219	-6,729	-4,271	-9,186	5,694	-19,151
42	6	2	66,249	70,019	68,134	3,770	-6,729	-4,271	-9,186	5,694	-19,151
8	9	2	80,336	61,199	70,768	-19,137	-6,729	-4,271	-9,186	5,694	-19,151
9	9	2	72,293	71,652	71,973	-0,641	-6,729	-4,271	-9,186	5,694	-19,151
22	12	2	77,666	71,937	74,802	-5,729	-6,729	-4,271	-9,186	5,694	-19,151
23	12	2	85,863	76,365	81,114	-9,498	-6,729	-4,271	-9,186	5,694	-19,151
24	12	2	71,561	63,362	67,462	-8,199	-6,729	-4,271	-9,186	5,694	-19,151
57	6	3	86,694	68,364	77,529	-18,330	-6,729	-4,271	-9,186	5,694	-19,151
58	6	3	80,647	82,277	81,462	1,630	-6,729	-4,271	-9,186	5,694	-19,151

S3 Table 2, continued.

**Animal No.**	**Time [wk]**	**Group**	rDW_{MRI} [%]	rDW_{Histo} [%]	$\left(\frac{rDW_{MRI}+rDW_{Histo}}{2}\right)$ [%]	$rDW_{Histo} - rDW_{MRI}$ [%]	$\bar{d}_{rDW}$ [%]	$\bar{d} + 95\,\%\,CI$ [%]	$\bar{d} - 95\,\%\,CI$ [%]	$\bar{d} + 1.96s_D$ [%]	$\bar{d} - 1.96s_D$ [%]
73	9	3	68,752	69,640	69,196	0,888	-6,729	-4,271	-9,186	5,694	-19,151
74	9	3	85,411	79,356	82,384	-6,055	-6,729	-4,271	-9,186	5,694	-19,151
45	12	3	83,621	73,252	78,437	-10,369	-6,729	-4,271	-9,186	5,694	-19,151
47	12	3	79,163	74,012	76,588	-5,151	-6,729	-4,271	-9,186	5,694	-19,151
48	12	3	82,297	74,517	78,407	-7,780	-6,729	-4,271	-9,186	5,694	-19,151
64	6	4	73,290	63,931	68,611	-9,359	-6,729	-4,271	-9,186	5,694	-19,151
65	6	4	68,905	67,481	68,193	-1,424	-6,729	-4,271	-9,186	5,694	-19,151
80	9	4	70,303	68,158	69,231	-2,145	-6,729	-4,271	-9,186	5,694	-19,151
82	9	4	86,924	82,675	84,800	-4,249	-6,729	-4,271	-9,186	5,694	-19,151
49	12	4	83,303	77,674	80,489	-5,629	-6,729	-4,271	-9,186	5,694	-19,151
50	12	4	86,633	88,608	87,621	1,975	-6,729	-4,271	-9,186	5,694	-19,151
51	12	4	82,051	81,619	81,835	-0,432	-6,729	-4,271	-9,186	5,694	-19,151

Bland-Altman plot

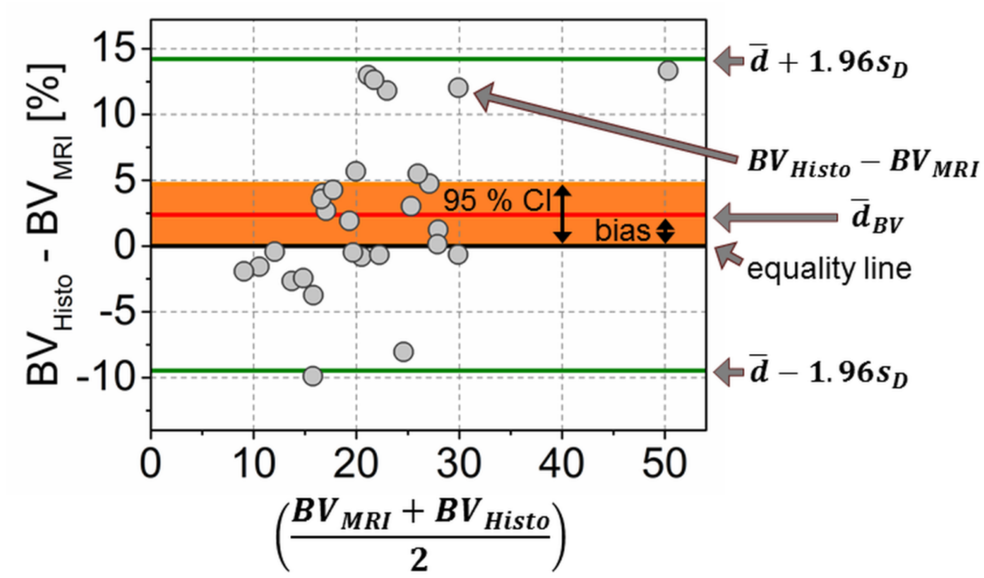

S3 Fig. 1 Bland-Altman diagram including the 95 % CI of the bias.
Because the 95 % CI [0,025 14,206] slightly excludes the line of equality, a significant bias of the measured values from MRI and histomorphometry can be assumed.
