# Supplementary material for: Assessing agreement between preclinical magnetic resonance imaging and histology: An evaluation of their image qualities and quantitative results
Source: PLoS One. 2017 Jun 30;12(6):e0179249. doi: 10.1371/journal.pone.0179249 (PMC5493293; doi:10.1371/journal.pone.0179249)
Supplement: S4 Appendix — (PDF) [file pone.0179249.s004.pdf]

S4 Appendix: Repeated measurements – Influence of the slice position

Descriptive statistics

S4 Table 1 Descriptive analysis of the repeated measurements for MRI, histomorphometry and their measurement differences. The single values can be found in the two tables for Bland-Altman analysis.

Data set	Parameter	d [%]	s_D [%]	SEM [%]	95 % CI [%]	d – 95 % CI [%]	d + 95 % CI [%]	Min [%]	Max [%]	Range [%]
MRT	*BV*	23,608	3,072	0,687	1,438	22,170	25,045	17,288	29,699	12,411
Histo	*BV*	22,018	1,978	0,442	0,926	21,093	22,944	18,709	25,606	6,897
Histo-MRT	*BV*	-1,589	3,450	0,772	1,615	-3,204	0,026	-9,001	5,193	14,195
MRT	*rDW*	69,002	1,574	0,352	0,736	68,266	69,739	66,332	72,821	6,489
Histo	*rDW*	68,346	0,651	0,146	0,305	68,041	68,650	67,225	70,061	2,836
Histo-MRT	*rDW*	-0,657	1,386	0,310	0,649	-1,305	-0,008	-4,389	1,349	5,738

d… mean value, s_D… standard deviation; SEM… standard error of the mean; CI… confidence interval

Bland-Altman analysis

S4 Table 2 Single values and Bland-Altman analysis of the repeated measurements, BV.

Measurement No.	BV_{MRI} [%]	BV_{Histo} [%]	$\left(\frac{BV_{MRI}+BV_{Histo}}{2}\right)$ [%]	$BV_{Histo} - BV_{MRI}$ [%]	$\bar{d}_{BV}$ [%]	$\bar{d} + 95\,\%\,CI$ [%]	$\bar{d} - 95\,\%\,CI$ [%]	$\bar{d} + 1.96s_D$ [%]	$\bar{d} - 1.96s_D$ [%]
1	21,619	21,755	21,687	0,136	-1,589	0,026	-3,204	5,173	-8,352
2	24,551	25,606	25,078	1,054	-1,589	0,026	-3,204	5,173	-8,352
3	19,577	22,508	21,043	2,931	-1,589	0,026	-3,204	5,173	-8,352
4	23,725	24,027	23,876	0,302	-1,589	0,026	-3,204	5,173	-8,352
5	27,096	23,132	25,114	-3,964	-1,589	0,026	-3,204	5,173	-8,352
6	22,783	20,995	21,889	-1,787	-1,589	0,026	-3,204	5,173	-8,352
7	25,771	18,709	22,240	-7,062	-1,589	0,026	-3,204	5,173	-8,352
8	20,318	22,376	21,347	2,058	-1,589	0,026	-3,204	5,173	-8,352
9	21,585	20,517	21,051	-1,068	-1,589	0,026	-3,204	5,173	-8,352
10	22,400	20,310	21,355	-2,090	-1,589	0,026	-3,204	5,173	-8,352
11	27,881	18,880	23,380	-9,001	-1,589	0,026	-3,204	5,173	-8,352
12	22,743	19,002	20,872	-3,741	-1,589	0,026	-3,204	5,173	-8,352
13	27,990	22,798	25,394	-5,192	-1,589	0,026	-3,204	5,173	-8,352
14	23,684	22,482	23,083	-1,202	-1,589	0,026	-3,204	5,173	-8,352

S4 Table 2, continued.

Measurement No.	BV_{MRI} [%]	BV_{Histo} [%]	$\left(\frac{BV_{MRI}+BV_{Histo}}{2}\right)$ [%]	$BV_{Histo} - BV_{MRI}$ [%]	$\bar{d}_{BV}$ [%]	$\bar{d} + 95\,\%\,CI$ [%]	$\bar{d} - 95\,\%\,CI$ [%]	$\bar{d} + 1.96s_D$ [%]	$\bar{d} - 1.96s_D$ [%]
15	29,699	25,022	27,360	-4,677	-1,589	0,026	-3,204	5,173	-8,352
16	24,520	20,590	22,555	-3,930	-1,589	0,026	-3,204	5,173	-8,352
17	17,288	22,482	19,885	5,193	-1,589	0,026	-3,204	5,173	-8,352
18	20,807	21,400	21,103	0,592	-1,589	0,026	-3,204	5,173	-8,352
19	24,153	23,631	23,892	-0,523	-1,589	0,026	-3,204	5,173	-8,352
20	23,960	24,146	24,053	0,186	-1,589	0,026	-3,204	5,173	-8,352

S4 Table 3 Single values and Bland-Altman analysis of the repeated measurements, *rDW*.

Measurement No.	rDW_{MRI} [%]	rDW_{Histo} [%]	$\left(\frac{rDW_{MRI}+rDW_{Histo}}{2}\right)$ [%]	$rDW_{Histo} - rDW_{MRI}$ [%]	$\bar{d}_{rDW}$ [%]	$\bar{d} + 95\,\%\,CI$ [%]	$\bar{d} - 95\,\%\,CI$ [%]	$\bar{d} + 1.96s_D$ [%]	$\bar{d} - 1.96s_D$ [%]
1	70,202	70,061	70,132	-0,141	-0,657	-0,008	-1,305	2,060	-3,373
2	72,821	68,432	70,626	-4,389	-0,657	-0,008	-1,305	2,060	-3,373
3	71,282	68,490	69,886	-2,792	-0,657	-0,008	-1,305	2,060	-3,373
4	69,388	68,788	69,088	-0,600	-0,657	-0,008	-1,305	2,060	-3,373
5	68,812	68,656	68,734	-0,156	-0,657	-0,008	-1,305	2,060	-3,373
6	71,066	68,505	69,785	-2,561	-0,657	-0,008	-1,305	2,060	-3,373
7	68,657	68,684	68,670	0,027	-0,657	-0,008	-1,305	2,060	-3,373

S4 Table 3, continued.

Measurement No.	rDW_{MRI} [%]	rDW_{Histo} [%]	$\left(\frac{rDW_{MRI}+rDW_{Histo}}{2}\right)$ [%]	$rDW_{Histo} - rDW_{MRI}$ [%]	$\bar{d}_{rDW}$ [%]	$\bar{d} + 95\,\%\,CI$ [%]	$\bar{d} - 95\,\%\,CI$ [%]	$\bar{d} + 1.96 s_D$ [%]	$\bar{d} - 1.96 s_D$ [%]
8	69,192	67,891	68,541	-1,301	-0,657	-0,008	-1,305	2,060	-3,373
9	69,347	67,872	68,609	-1,475	-0,657	-0,008	-1,305	2,060	-3,373
10	69,744	67,991	68,867	-1,752	-0,657	-0,008	-1,305	2,060	-3,373
11	67,822	67,533	67,677	-0,289	-0,657	-0,008	-1,305	2,060	-3,373
12	67,839	68,308	68,073	0,468	-0,657	-0,008	-1,305	2,060	-3,373
13	67,980	68,603	68,291	0,622	-0,657	-0,008	-1,305	2,060	-3,373
14	68,657	67,982	68,319	-0,675	-0,657	-0,008	-1,305	2,060	-3,373
15	67,839	68,546	68,192	0,706	-0,657	-0,008	-1,305	2,060	-3,373
16	67,822	67,815	67,818	-0,007	-0,657	-0,008	-1,305	2,060	-3,373
17	66,667	67,225	66,946	0,558	-0,657	-0,008	-1,305	2,060	-3,373
18	66,332	67,681	67,006	1,349	-0,657	-0,008	-1,305	2,060	-3,373
19	70,051	69,447	69,749	-0,603	-0,657	-0,008	-1,305	2,060	-3,373
20	68,528	68,404	68,466	-0,124	-0,657	-0,008	-1,305	2,060	-3,373
